# FBLN7 KO attenuates age-related cardiac fibrosis by promoting TGFBR3/ALK1/Smad1 signaling and inhibiting the profibrotic phenotypes of cardiac fibroblasts

**DOI:** 10.7150/thno.116477

**Published:** 2025-07-28

**Authors:** Xuehui Zheng, Guoqing Yao, Huaitao Yu, Binghui Kong, Yuan Zhao, Yang Hu, Xiangping Ma, Jinghan Hai, Panpan Xu, Yun Ti, Peili Bu

**Affiliations:** State Key Laboratory for Innovation and Transformation of Luobing Theory; Key Laboratory of Cardiovascular Remodeling and Function Research of MOE, NHC, CAMS and Shandong Province; Department of Cardiology, Qilu Hospital of Shandong University, Jinan, China, 250012.

**Keywords:** FBLN7, cardiac fibrosis, fibroblasts senescence, TGFBR3, natural products

## Abstract

**Rationale:** Aging induces structural and functional changes in the heart, including left ventricular (LV) hypertrophy, a decline in diastolic function, and even heart failure. Fibulin 7 (FBLN7) is a key mediator of extracellular matrix (ECM) remodeling under pathological conditions. In our study, we aim to explore whether FBLN7 is also involved in the development of age-related cardiac fibrosis and its underlying mechanisms.

**Methods:** We generated naturally aged FBLN7 knockout and wild-type mice (18 months old). Western blot and immunofluorescence assays were employed to investigate the biological function of FBLN7 in senescent cardiac fibroblasts. The interaction between FBLN7 and cell membrane receptors was explored through molecular docking and co-immunoprecipitation techniques. The interaction between FBLN7 and natural products was explored through virtual screening, molecular dynamics simulations and surface plasmon resonance (SPR).

**Results:** Our results demonstrated that the cardioprotective effects observed in aged FBLN7 knockout (KO) mice are mediated by the inhibition of profibrotic phenotypes in senescent cardiac fibroblasts (CFs), which reduces age-related myocardial fibrosis and ultimately improves cardiac diastolic function. The observation that overexpressing FBLN7 in fibroblast-specific protein 1 positive (FSP1^+^) cells of aged mice exacerbates age-related myocardial fibrosis further supports this finding. Mechanistically, we identified that FBLN7 promotes the proliferation, migration, actin remodeling, and collagen production of senescent CFs at least partially by binding to TGFBR3 and reducing its protein levels, thereby inhibiting the activation of the ALK1-Smad1/5/9 pathway. Additionally, we identified a natural product, Ginsenoside Ro, that physically interacts with FBLN7 and validated its antifibrotic activity both in vitro and in vivo.

**Conclusions:** These findings reveal FBLN7 reverses the impaired profibrotic phenotypes of senescent CFs, thereby aggravating age-related cardiac fibrosis. Given that age-related fibrosis is a significant pathological factor in heart failure with preserved ejection fraction (HFpEF), downregulating FBLN7 and/or interfering with its function may represent an effective therapeutic strategy for HFpEF.

## Introduction

Aging is recognized as one of the most significant cardiovascular risk factors 1. The prevalence of individuals with diastolic dysfunction or even heart failure markedly increases with advancing age 2. Aging induces cardiac structural and functional changes, including left ventricular (LV) hypertrophy, a decline in diastolic function, and even heart failure, are significant concerns in aging-related heart disease 3, 4. Aging-related heart disease is characterized by heart failure with preserved ejection fraction (HFpEF) caused by cardiac fibrosis, which results from increased deposition of extracellular matrix (ECM) protein, and has been shown to have clinical importance 5-7. Recent studies have underscored the importance of matricellular proteins in the interplay between cellular senescence, collagen deposition, and cell death 8. Therefore, the roles of matricellular proteins in aging-related cardiac fibrosis warrant further investigation.

Fibulin 7 (FBLN7) is a cell-adhesive protein derived from the extracellular matrix (ECM) that plays a crucial role in creating a microenvironment conducive to various cellular functions by regulating cell differentiation, migration, and adhesion 9-11. Our recent studies have demonstrated that FBLN7 is minimally expressed in healthy myocardium but is significantly upregulated following cardiac injury. This upregulation is associated with pronounced interstitial and perivascular fibrosis, which contributes to the development of heart failure 11. Furthermore, FBLN7 promotes pressure-induced vascular remodeling by modifying smooth muscle cell phenotypes and ECM deposition 12. Therefore, FBLN7 serves as a key mediator of ECM remodeling under pathological conditions. However, it remains unclear whether FBLN7 is also implicated in the development of age-related cardiac dysfunction. Building on previous findings, we aimed to investigate the potential role of FBLN7 in cardiac fibrosis and diastolic dysfunction within the context of aging.

## Results

### FBLN7 expression is increased in the aging heart

**Figure 1:**
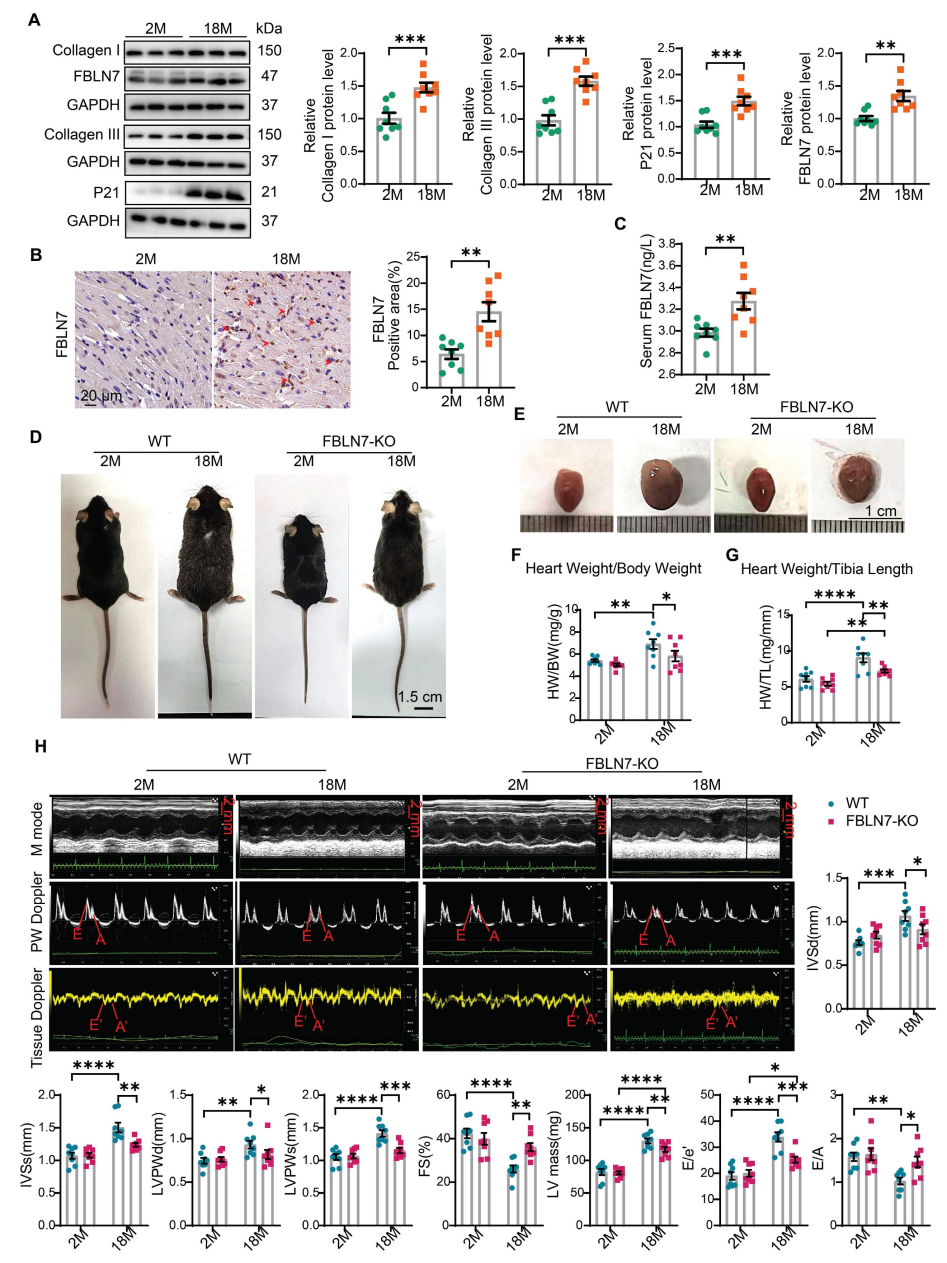
** Loss of FBLN7 exacerbates age-related cardiac hypertrophy and diastolic dysfunction.** A) Representative Western blot images and quantification of Collagen I, Collagen III, FBLN7, and P21 protein levels in the hearts of wild-type (WT) mice at 2 months and 18 months of age. Western blot analyses show the expression of these proteins, with 8 mice in each group. B) Representative images of immunohistochemical staining for FBLN7 in heart sections from WT mice at 2 months and 18 months, with quantification displayed on the right. C) ELISA detection of serum FBLN7 levels. D) Gross appearance of WT mice and FBLN7 knockout (KO) mice at 2 months and 18 months of age. E) Gross appearance of the hearts of WT mice and FBLN7-KO mice at 2 months and 18 months of age. F) Heart weight normalized to body weight (HW/BW) of the indicated groups of mice. G) Heart weight normalized to tibia length (HW/TL) of the indicated groups of mice. H) Representative echocardiography images of the four groups of mice. Upper: B-mode; middle: PW Doppler mode; lower: tissue Doppler mode. Echocardiographic analysis of interventricular septal thickness in diastole (IVSd), interventricular septal thickness in systole (IVSs), left ventricular posterior wall thickness in diastole (LVPWd), left ventricular posterior wall thickness in systole (LVPWs), fractional shortening (FS%), LV mass, the ratio of early diastolic filling velocity to left atrial pressure (E/e') and the ratio of early to late diastolic filling velocities (E/A) are presented on the right and bottom. Error bars represent the standard error of the mean (SEM). *P < 0.05, **P < 0.01, ***P < 0.001, ****P < 0.0001.

To investigate the role of FBLN7 in age-related cardiac fibrosis, we first assessed its expression levels in aging hearts. We found that the P21 protein level was significantly elevated in aged mice (18 months old) compared to young mice (2 months old), indicating cardiac aging. Additionally, the protein levels of Collagen I (Col I) and Collagen III (Col III) were significantly increased in older mice, contributing to cardiac fibrosis, when compared to the young group. Notably, the protein level of FBLN7 was also significantly elevated in aging hearts (Figure 1A). This increase in FBLN7 was similarly observed in both naturally aging hearts (Figure 1B) and in D-galactose (D-gal)-induced aging hearts (Figure S1A), as detected by immunohistochemistry (IHC). Given that FBLN7 is a secreted protein, we also measured serum FBLN7 levels in aged mice and found a significant increase compared to young mice (Figure 1C). These findings demonstrate that FBLN7 expression is associated with age-related cardiac fibrosis.

### FBLN7 KO alleviates age-related cardiac fibrosis and diastolic dysfunction

**Figure 2:**
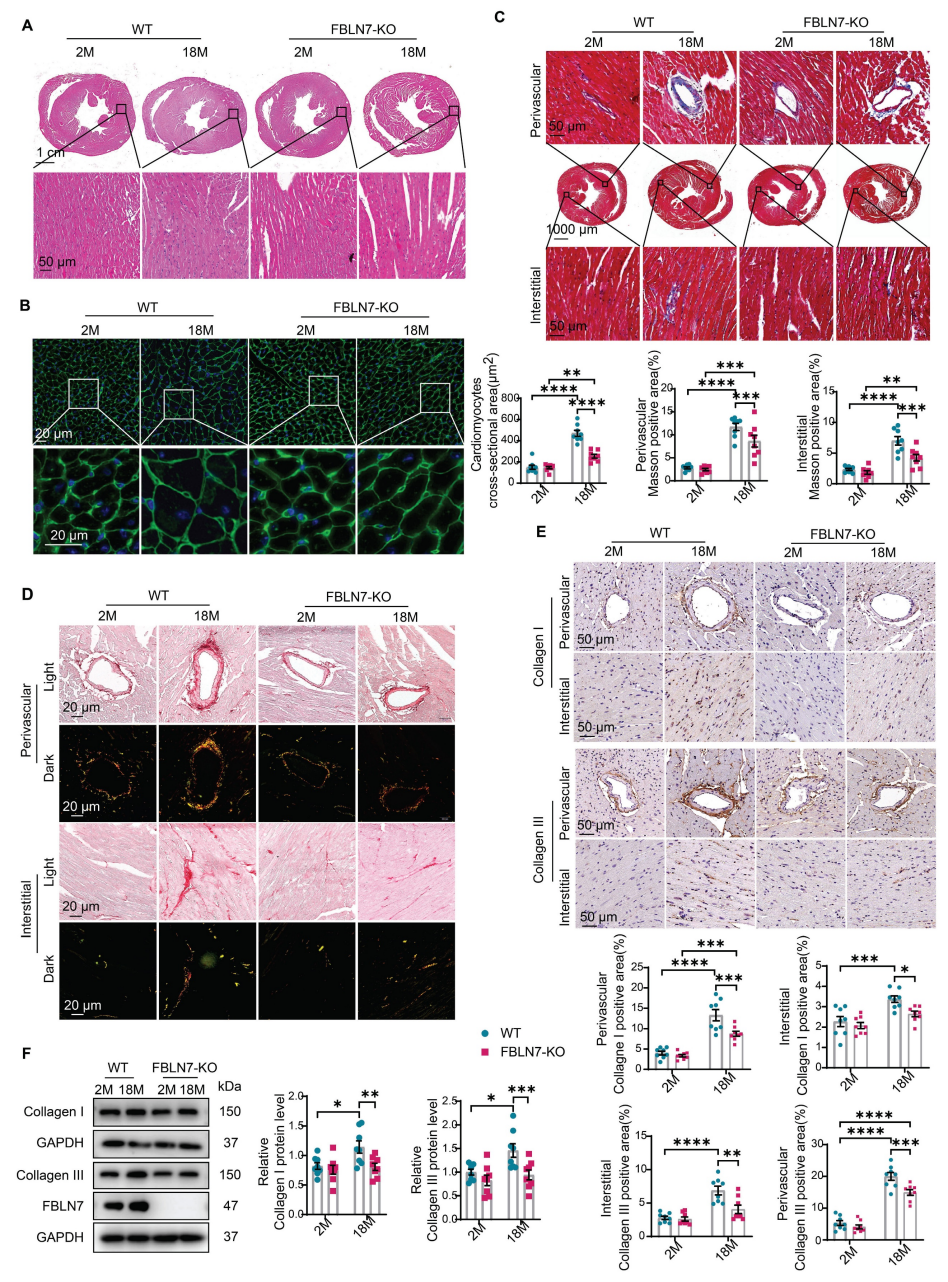
** FBLN7 KO protects mice against age-related cardiac fibrosis.** Cardiac fibrosis was assessed in wild-type (WT) and FBLN7 knockout (KO) mice at 2 months and 18 months of age. A) Representative images of hematoxylin and eosin (H&E) staining of hearts from the four groups of mice. The lower panel shows the enlargement of the black box. B) Representative images of wheat germ agglutinin (WGA) staining for the four groups of mice, with quantifications displayed around. The lower panel shows the enlargement of the white box. C) Representative micrographs of Masson's trichrome staining in heart sections from the four groups of mice, showing three different views: upper (perivascular), middle (global), and lower (interstitial). Quantifications of fibrotic areas are presented at the bottom. D) Representative images of heart sections stained with Sirius red. E) Representative micrographs of immunohistochemical (IHC) staining for Collagen I and III, along with corresponding statistical plots shown below. Both perivascular and interstitial views are included. F) Representative western blot images and quantifications of collagen I, collagen III and FBLN7 protein levels are presented. Error bars are SEM. *P < 0.05, **P < 0.01, ***P < 0.001, ****P < 0.0001.

To further investigate the role of FBLN7, we generated FBLN7-deficient mice (FBLN7-KO). The FBLN7-KO mice were born at the expected frequency and developed normally for at least 18 months. Both gross and microscopic morphology appeared normal in these mice, and no developmental abnormalities were observed. Additionally, FBLN7 was not detected in the heart tissues in FBLN7-KO mice (Figure 2F).

To determine the role of FBLN7 in age-related cardiac fibrosis, we conducted a study using naturally aged FBLN7 knockout (FBLN7-KO) mice at 18 months of age (Figure 1D). The explanted hearts from the FBLN7-KO-18M mice appeared smaller compared to those from the wild-type (WT)-18M mice (Figure 1E). The heart-to-body weight ratios (HW/BW) and the heart weight to tibia length ratios (HW/TL) of the WT-18M mice exhibited an increase in heart mass relative to the FBLN7-KO-18M group (Figures 1F and G). In contrast to the WT-18M group, the FBLN7-KO-18M group demonstrated improved left ventricular (LV) relaxation, as indicated by an increase in E/A ratios and decrease in E/e', and reduced LV hypertrophy, as evidenced by decreases in the interventricular septum thickness at end diastole (IVSd) and systole (IVSs), LV posterior wall thickness at diastole (LVPWd) and systole (LVPWs), and LV mass, all detected via echocardiography (Figure 1H). However, analysis of LV ejection fraction (LVEF), as well as LV internal diameters at end systole (LVIDs) and diastole (LVIDd), revealed no significant restoration of LV dimensions following FBLN7 KO (Figure S1B). Furthermore, hematoxylin and eosin (HE) and wheat germ agglutinin (WGA) staining of heart sections demonstrated reduced cardiac hypertrophy and a decrease in cardiomyocyte cross-sectional area after FBLN7 deletion (Figure 2A and B). We subsequently measured cardiac fibrosis, another hallmark of age-related structural remodeling in the human heart and decline in heart function 13. Masson's trichrome staining revealed increased collagen deposition in the interstitial space and perivascular regions of the hearts from 18M mice, while FBLN7 deletion resulted in a reduction of collagen deposition area (Figure 2C). Polarized light microscopy of Picro-Sirius Red-stained heart sections showed decreases in the areas of Col I (red-yellow fibers) and Col III (green fibers) in the interstitial space and perivascular regions of aged FBLN7-KO mice compared to aged WT mice (Figure 2D). This finding was consistent with the changes in Col I and Col III protein levels detected by IHC (Figure 2E) and WB (Figure 2F). These data indicate that the deletion of FBLN7 attenuates myocardial fibrosis, LV hypertrophy, and diastolic dysfunction during cardiac aging.

### CFs may contribute to cardioprotective effects observed in aged FBLN7-KO mice

**Figure 3:**
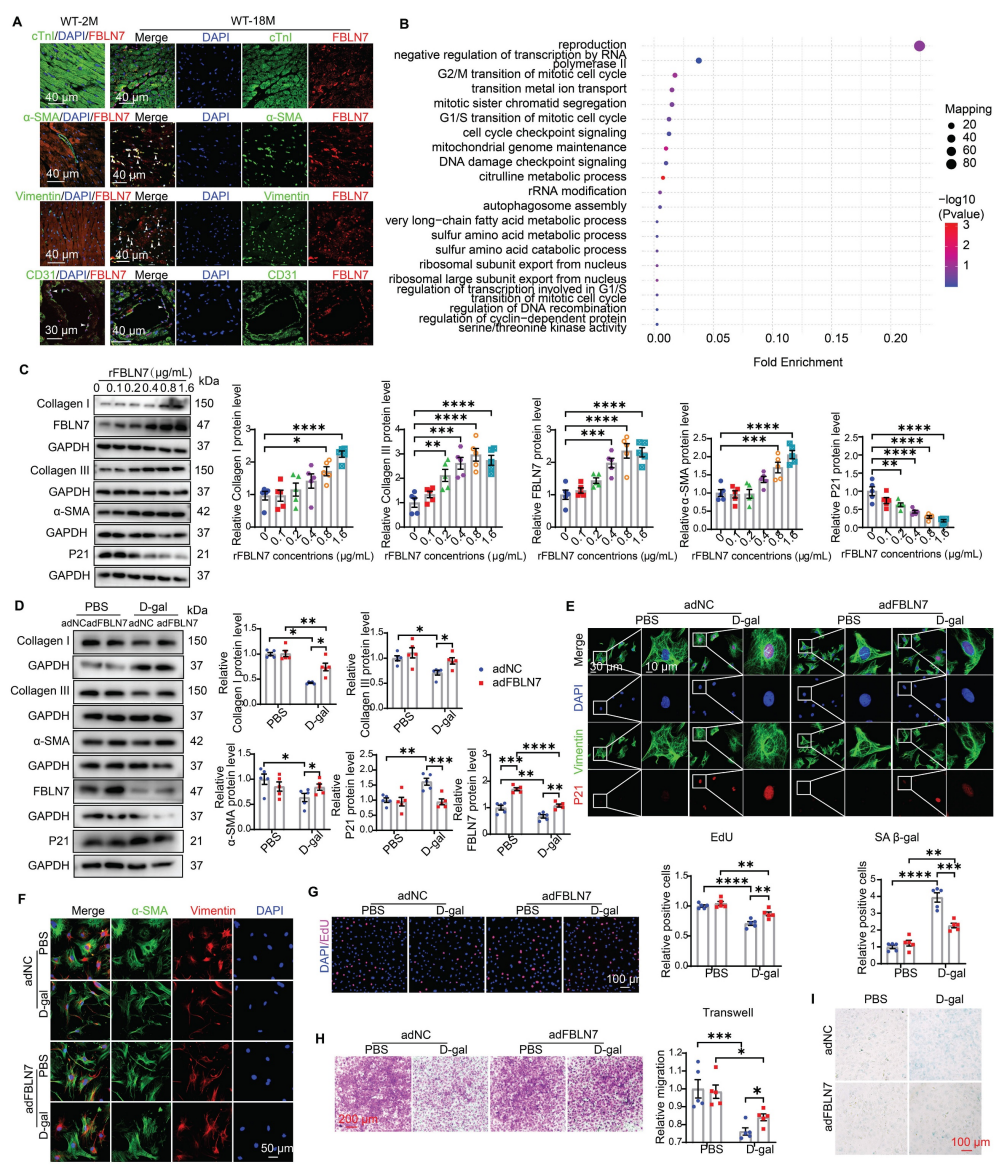
** FBLN7 reversed the impaired profibrotic phenotypes of senescent CFs in vitro.** A) Representative Immunofluorescent (IF) micrographs illustrating the co-localization of FBLN7 with cTnI, α-SMA, Vimentin, and CD31 in wild-type aging hearts (at 18 months of age) and young hearts (at 2 months of age) are presented. White arrowheads indicate co-localization (yellow). B) Ontology (GO) term analysis (biological processes) of differentially expressed proteins in the left ventricular tissues of FBLN7 gene knockout mice and wild-type mice. C) Representative Western blot images depict the expression of Collagen I, Collagen III, FBLN7, α-SMA, and P21 proteins in primary cardiac fibroblasts (CFs) treated with various concentrations of recombinant FBLN7 protein. Quantifications are shown on the right. D-I. The reversal effects of FBLN7 overexpression (adFBLN7) on the impaired profibrotic phenotypes of senescent CFs induced by D-galactose (D-gal) are illustrated. D) Representative Western blot images show the expression of Collagen I and III, α-SMA, P21, and FBLN7 proteins in FBLN7-overexpressing CFs after treatment with D-gal or PBS. Quantifications are displayed on the right. E) Representative images depict P21 expression (red) in CFs labeled with Vimentin (green). F) Representative IF images of CFs stained with α-SMA (green) and Vimentin (red), with nuclei stained using DAPI (blue), are shown. G) Representative images of CFs co-stained with EdU (red) and DAPI (blue) are presented, with the quantification of EdU-positive cells indicated on the right. H) Representative photomicrographs from Transwell assays are included, with the quantification of migrating CFs shown on the right. I) Representative images of senescence-associated β-galactosidase staining are provided, along with the quantification of β-galactosidase-positive cells (blue-green) displayed above the image. Error bars represent the standard error of the mean (SEM). Statistical significance is indicated as follows: *P < 0.05, **P < 0.01, ***P < 0.001, ****P < 0.0001.

To determine which cell type contributes to the protective effect of FBLN7 knockout, we first examined the distribution of FBLN7 through immunofluorescence (IF) staining in aging hearts. The results revealed that FBLN7 was predominantly found in endothelial cells (ECs) in young hearts (Figure 3A). In aging hearts, in addition to ECs, FBLN7 co-localized primarily with cardiac fibroblasts (CFs) and myofibroblasts (MCFs), while exhibiting minimal co-localization with cardiomyocytes (CMs) (Figure 3A). This indicates that the changes in FBLN7 expression are mainly driven by CFs and MCFs. Furthermore, data from a single-cell transcriptomic analysis of primate cardiac aging, obtained from public databases, indicate that Fbln7 is predominantly differentially expressed in CFs (P=2.82E-240) and MCFs (P=3.07E-07) (Figure S1C). The differences observed in CFs were particularly pronounced. These findings suggest that the accumulation of FBLN7 in the aging heart may promote collagen deposition via CFs, as CFs are the primary source of collagen in the heart.

We also conducted a proteomic analysis in left ventricular cardiac tissue from WT and FBLN7-KO aging mouse hearts (18 months old). Gene Ontology (GO) term analysis revealed that the differentially expressed (DE) proteins were primarily associated with the cell cycle (Figure 3B). Kyoto Encyclopedia of Genes and Genomes (KEGG) pathway analysis indicated that these DE proteins were enriched in multiple pathways, including the MAPK signaling pathway, transcriptional dysregulation in cancer and gastric cancer, and cytokine-cytokine receptor interactions, many of which are related to cellular senescence (Figure S1D). This suggests a possible link between FBLN7 and CF senescence, considering the localization of FBLN7 in aging hearts.

To define the role of FBLN7 in CF senescence and collagen synthesis, we isolated primary CFs and treated them with various concentrations of recombinant FBLN7 protein (rFBLN7). The results demonstrated a trend of increasing levels of Col I, Col III, and alpha-smooth muscle actin (α-SMA), alongside a decrease in the levels of the P21 protein, in correlation with rising concentrations of rFBLN7. This indicates the profibrotic and anti-senescent roles of FBLN7 in CFs (Figure 3C). Collectively, the protective function of FBLN7 deletion in age-related cardiac fibrosis and diastolic dysfunction may be attributed to the loss of its profibrotic and anti-senescent effects on CFs.

### FBLN7 reversed the impaired profibrotic phenotypes of senescent CFs

To determine whether the profibrotic and anti-aging effects of FBLN7 on CFs are altered in the context of aging, we employed an in vitro model of senescent cells induced by D-galactose (D-gal). Compared to young CFs treated with phosphate-buffered saline (PBS), CFs treated with D-gal exhibited elevated senescence markers, such as P21 expression (Figures 3D and E, Figures S1G and H) and senescence-associated β-galactosidase (SA β-gal) activity (Figure 3I), indicating CF senescence. Concurrently, abilities related to fibrosis, including proliferation (Figure 3G), migration (Figure 3H), actin remodeling (α-SMA expression) (Figures 3D and F), and collagen production (Figure 3D), decline. Thus, senescent CFs displayed impaired profibrotic phenotypes. We next tested whether the overexpression or reduced expression of FBLN7 would affect these phenotypes of senescent CFs. WB and ELISA were performed to verify the silencing or overexpression efficiency of FBLN7 (Figures 3D, Figures S1E-G). Our findings revealed that FBLN7 overexpression reverses the impaired profibrotic phenotypes of senescent CFs, as indicated by enhanced proliferation and migration capacities (Figures 3G and H) and elevated levels of Col I, Col III, and α-SMA proteins (Figures 3D and F) compared to control senescent CFs. Additionally, FBLN7 overexpression inhibited senescence in CFs induced by D-gal, as evidenced by decreased levels of P21 protein (Figures 3D and E) and reduced SA β-gal activity (Figure 3I). In contrast, silencing FBLN7 further exacerbated senescence and the impaired profibrotic phenotypes of CFs (Figures S1G-L). These data suggest that FBLN7 reverses the impaired profibrotic phenotypes of senescent CFs.

### FBLN7 modulates impaired profibrotic abilities of senescent CFs through ALK1-Smad1/5/9 pathway

**Figure 4:**
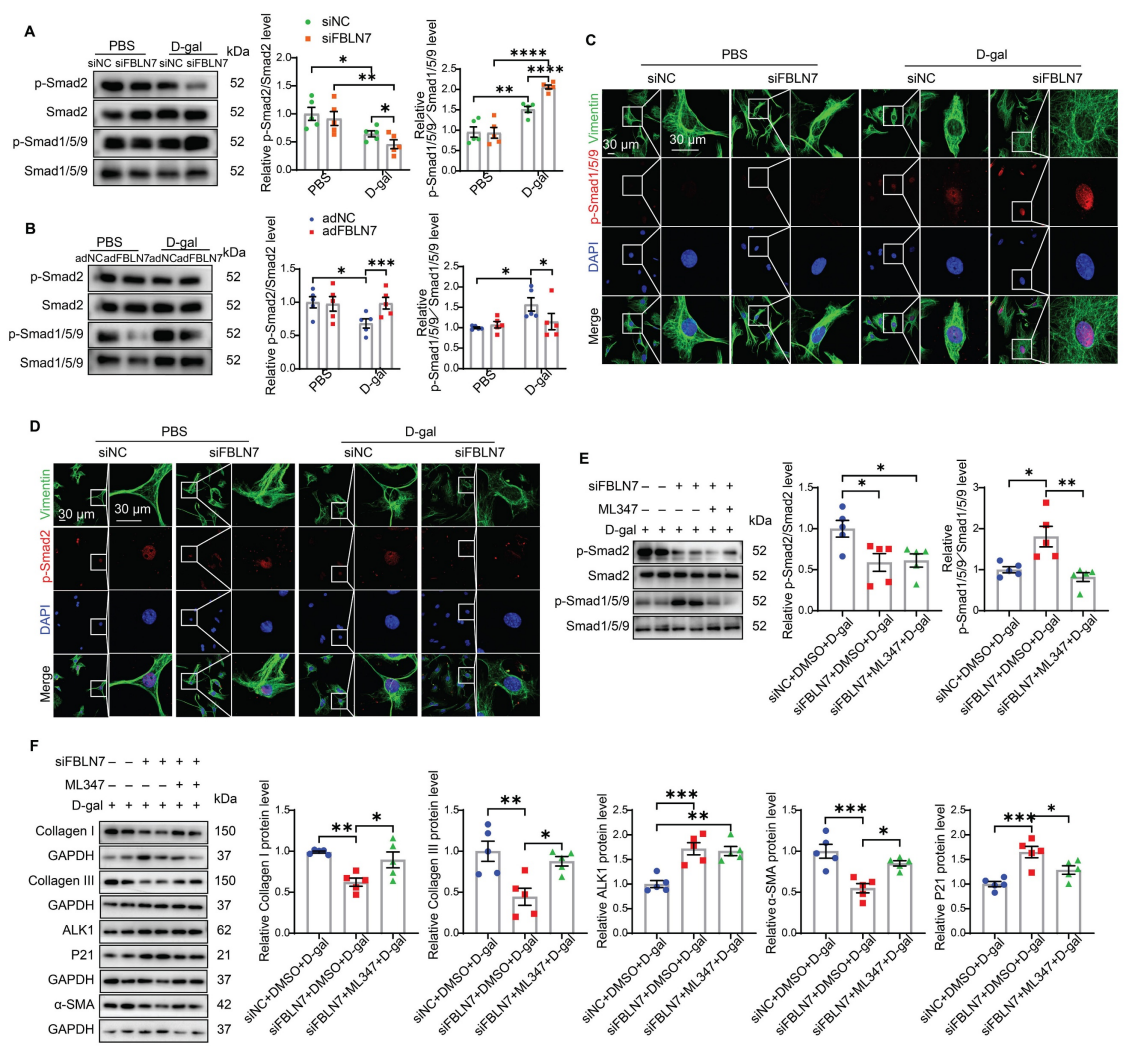
** FBLN7 modulates the impaired profibrotic abilities of senescent cardiac fibroblasts through the ALK1-Smad1/5/9 pathway.** A-B) Representative western blot images displaying the levels of p-Smad2, Smad2, p-Smad1/5/9, and Smad1/5/9 proteins in cardiac fibroblasts with FBLN7 silencing (siFBLN7) (A), FBLN7 overexpression (adFBLN7) (B), and corresponding control cardiac fibroblasts (siNC or adNC) following treatment with D-gal or PBS. Quantifications of band intensity are presented on the right. C-D) Representative images illustrating the nuclear translocation of p-Smad1/5/9 (red) (C) and p-Smad2 (red) (D) in siFBLN7 and siNC cardiac fibroblasts after treatment with D-gal or PBS, labeled with Vimentin (green). E) Representative western blot images showing the protein levels of p-Smad2, Smad2, p-Smad1/5/9 and Smad1/5/9 in D-gal treated cardiac fibroblasts transfected with siNC, siFBLN7, or siFBLN7 plus ML347 treatment. Quantifications are displayed on the right. F) Representative western blot images showing the protein levels of Collagen I and III, ALK1, α-SMA and P21 in D-gal treated cardiac fibroblasts transfected with siNC, siFBLN7, or siFBLN7 plus ML347 treatment. Quantifications are displayed on the right. Error bars represent the standard error of the mean (SEM). Statistical significance is indicated as follows: *P < 0.05, **P < 0.01, ***P < 0.001, ****P < 0.0001.

The TGF-β signaling pathway is the most significant pathway involved in myocardial fibrosis and is one of the key pathways regulating both damage-induced senescence and developmentally programmed senescence 14, 15. It ranks among the top upregulated pathways observed across various cell types in aging hearts 16. Activated TGF-β signaling can phosphorylate two Smad signaling pathways: the anti-fibrotic Smad1/5 pathway (via activin receptor-like kinase 1, ALK1) and the pro-fibrotic Smad2/3 pathway (via ALK5) during progressive fibrosis 17. Therefore, we started by focusing on these two signaling pathways in senescent CFs. Young CFs exhibited low levels of Smad1/5/9 phosphorylation and nuclear translocation, whereas these levels were significantly increased in senescent CFs (Figure 4 A-C). In contrast, Smad2 phosphorylation and nuclear translocation were markedly reduced in senescent CFs (Figure 4 A, B, and D). Silencing of FBLN7 further enhanced the activation of Smad1/5/9 (Figure 4 A and C) and inhibited Smad2 phosphorylation (Figure 4 A and D) in senescent CFs, while overexpression of FBLN7 produced the opposite effects (Figure 4 B and Figure S2 A and B). Thus, FBLN7 may differentially regulate TGF-β signal transduction in senescent CFs by augmenting the ALK5-Smad2 pathway and inhibiting the ALK1-Smad1/5/9 pathway.

The TGF-β1/ALK5/Smad2 signaling pathway has been extensively studied in the context of cardiac fibrosis, while the role of the ALK1/Smad1 signaling pathway remains less understood. Therefore, we focused on whether the ALK1-Smad1/5/9 pathway contributes to the impairment in profibrotic phenotypes of senescent CFs promoted by FBLN7 silencing. Given that reduced ALK1 expression appears to be associated with cardiac fibrosis 18, we hypothesized that FBLN7 may exert its effects through the ALK1-Smad1/5/9 pathway. To test this hypothesis, we introduced the ALK1 inhibitor ML347 in FBLN7-silenced senescent CFs, which significantly inhibited the phosphorylation of Smad1/5/9 (Figure S2C). The levels of FBLN7 in siFBLN7-transfected senescent CFs and cell culture supernatants were significantly reduced compared to the control group, indicating successful knockdown (Figure S2D and E). Our observations indicated that ML347 effectively suppressed the elevated phosphorylation of Smad1/5/9 induced by FBLN7 silencing, while having minimal impact on Smad2 phosphorylation and FBLN7 expression (Figure 4E and Figure S2D). Additionally, ML347 partially counteracted the promotion of impaired profibrotic phenotypes following FBLN7 knockdown in senescent CFs, as evidenced by increased levels of Col I, Col III, and α-SMA proteins after ML347 treatment in FBLN7-silenced senescent CFs (Figure 4F). These data suggest that FBLN7 exerts its effects partially through the ALK1-Smad1/5/9 pathway.

To further verify the critical role of the ALK1-Smad1/5/9 pathway in the pro-fibrotic function of FBLN7, we overexpressed ALK1 in FBLN7-overexpressing senescent CFs. The results indicated that Flag-ALK1 significantly restored the reduced phosphorylation of Smad1/5/9 induced by FBLN7 overexpression, while having minimal impact on Smad2 phosphorylation (Figure S3A). Additionally, Flag-ALK1 partially mitigated the profibrotic effects of FBLN7 overexpression on senescent CFs, as evidenced by decreased levels of Col I and Col III proteins (Figure S3B). Collectively, these findings suggest that FBLN7 may reverse the impaired profibrotic phenotypes of senescent CFs by differentially regulating TGF-β signal transduction, at least in part by inhibiting ALK1-mediated Smad1/5/9 activation.

### TGFBR3 is required for FBLN7-mediated differential transduction of TGF-β signals

**Figure 5:**
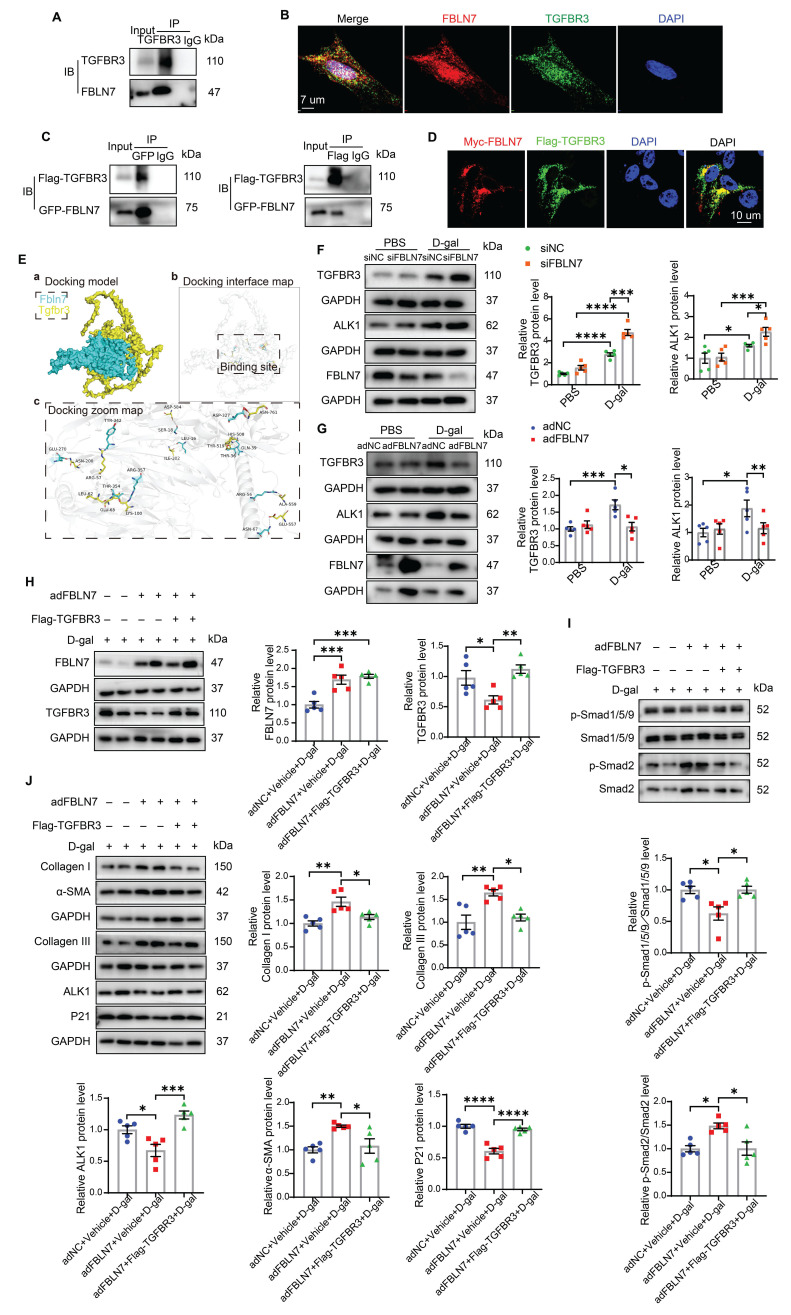
** TGFBR3 is essential for the differential transduction of TGF-β signals mediated by FBLN7.** A) Endogenous co-immunoprecipitation (Co-IP) of FBLN7 and TGFBR3. Immunoprecipitation (IP) was performed with TGFBR3, followed by immunoblotting (IB) with FBLN7 in senescent cardiac fibroblasts (CFs). B) Representative confocal images demonstrating the co-localization of FBLN7 and TGFBR3 in senescent CFs. C) Reciprocal Co-IP of FBLN7 and TGFBR3. IP was conducted with GFP-tag and IB with Flag-TGFBR3 in 293T cells (left panel). Conversely, IP was performed with Flag-tag and IB with GFP-FBLN7 in 293T cells (right panel). D) Representative confocal images illustrating the co-localization of Myc-FBLN7 and Flag-TGFBR3 in 293T cells. E) Results of molecular docking between FBLN7 and TGFBR3. a) The optimal docking conformation of FBLN7 and TGFBR3 reveals a stable complex, with FBLN7 highlighted in blue and TGFBR3 in yellow. b) The white semi-transparent structure represents the entire molecular framework, providing a global view of the docking region. The black box in the center indicates the binding site, emphasizing critical areas on the binding interface. c) The Docking Zoom Map displays key amino acid residues at the binding interface and their interactions. F-G) Representative western blot images showing TGFBR3 and ALK1 protein levels in CFs with FBLN7 silencing (F) and overexpression (G) after treatment with D-gal or PBS. Quantifications of band intensity are presented on the right. H) Representative western blot images illustrating protein levels of FBLN7 and TGFBR3 in D-gal-treated CFs infected with either Null, adenovirus encoding FBLN7 (adFBLN7), or adFBLN7 plus Flag-TGFBR3. Quantifications are displayed on the right. I) Representative western blot images illustrating protein levels of p-Smad1/5/9, Smad1/5/9, p-Smad2 and Smad2 in D-gal-treated CFs infected with either Null, adFBLN7, or adFBLN7 plus Flag-TGFBR3. Quantifications are displayed on the bottom. J) Representative western blot images illustrating protein levels of Collagen I and III, ALK1, α-SMA, and P21 in D-gal-treated CFs infected with either Null, adFBLN7, or adFBLN7 plus Flag-TGFBR3. Quantifications are displayed around the imge. Error bars represent the standard error of the mean (SEM). Statistical significance is indicated as follows: *P < 0.05, **P < 0.01, ***P < 0.001, ****P < 0.0001.

Considering that FBLN7 is an ECM protein, we first examined its interaction with three types of TGF-β receptors: TGF-β type I receptor (TGFBR1), TGF-β type II receptor (TGFBR2), and TGF-β type III receptor (TGFBR3), as well as ALK1. Co-immunoprecipitation (Co-IP) assays demonstrated that the immunoprecipitation (IP) of endogenous TGFBR3 resulted in the co-precipitation of FBLN7 in senescent CFs (Figure 5A). Endogenous FBLN7 was nearly undetectable in the IP of TGFBR1 and TGFBR2. Additionally, endogenous FBLN7 could not be identified in the immunoprecipitated ALK1 (Figure S4). These results suggest that FBLN7 may interact with TGFBR3 rather than TGFBR1, TGFBR2, or ALK1. The TGFBR3 serves as a co-receptor for the TGF-β superfamily. It facilitates the presentation of ligands to the TGF-β signaling receptors and plays a regulatory role in fibrosis 19, 20. TGFBR3 has been identified as a "switch" that attenuates ALK5-Smad2/3 signaling while enhancing ALK1-Smad1 signaling 19. Consequently, TGFBR3 may function as the membrane receptor that transmits the FBLN7 signal from the ECM to Smads.

To test this hypothesis, we further validated the interaction between FBLN7 and TGFBR3. IF and confocal microscopy images revealed that a substantial portion of TGFBR3 in senescent CFs was colocalized with FBLN7 (Figure 5B). We also constructed Flag-TGFBR3 and GFP-FBLN7 plasmids and co-transfected them into 293T cells. Similar results were obtained in reciprocal Co-IP assays and confocal IF imaging (Figures 5C and 5D), demonstrating that FBLN7 can interact with TGFBR3. Additionally, molecular docking was performed using GRAMM-X to simulate the interaction between TGFBR3 and FBLN7, further supporting the physical interaction between the two proteins (Figure 5E).

Changes in TGFBR3 protein levels also impact TGF-β signal transduction; therefore, we next assessed whether FBLN7 overexpression or silencing alters the protein level of TGFBR3 in senescent CFs. Compared to young CFs, senescent CFs express higher levels of TGFBR3. Silencing FBLN7 further upregulated TGFBR3 expression, while FBLN7 overexpression had the opposite effect (Figure 5F and G). Furthermore, we observed that the expression of ALK1 mirrored the changes in TGFBR3 expression: it was significantly upregulated in FBLN7-silenced senescent CFs but downregulated in those with FBLN7 overexpression (Figure 5F and G). Taken together, these findings suggest that FBLN7 likely acts by interfering TGFBR3 function.

To further investigate the involvement of TGFBR3 in the FBLN7-mediated negative regulation of the ALK1-Smad1/5/9 pathway, we overexpressed TGFBR3 using the Flag-TGFBR3 plasmid in FBLN7-overexpressing senescent CFs. The protein levels of FBLN7 and TGFBR3 in senescent CFs were significantly increased compared to the respective controls, demonstrating successful transfection (Figure 5H). At the same time, FBLN7 was measured in cell culture supernatants by ELISA and indicated successful overexpression (Figure S5A). We observed elevated levels of ALK1 and p-Smad1/5/9 (Figure 5I and J) and decreased levels of p-Smad2 (Figure 5I) in both FBLN7 and TGFBR3 overexpressing senescent CFs, compared to senescent CFs overexpressing FBLN7 alone. This indicates that FBLN7 mediates differential transduction of TGF-β signals at least partially by reducing TGFBR3 expression, thereby affecting ALK1 expression. Furthermore, TGFBR3 overexpression mitigated the profibrotic effects on senescent CFs caused by FBLN7 overexpression, as evidenced by decreased levels of Col I, Col III, and α-SMA proteins, along with increased levels of P21 protein following TGFBR3 overexpression (Figure 5J). To further verify the critical role of TGFBR3 in the pro-fibrotic function of FBLN7, we silenced TGFBR3 in FBLN7-silencing senescent CFs. The results indicated that TGFBR3-scilencing significantly inhibited the decreases in Col I, Col III, α-SMA and the increases in P21 induced by FBLN7-scilencing (Figure S5B). Additionally, TGFBR3-scilencing inhibited the decrease in p-Smad2 level and increase in p-Smad1/5/9 level induced by FBLN7-scilencing (Figure S5C). Therefore, TGFBR3 contributes to FBLN7-mediated inhibition of ALK1-Smad1/5/9 signaling and the acquisition of profibrotic phenotypes in senescent CFs.

### Overexpression of FBLN7 in FSP1^+^ cells promotes age-related cardiac fibrosis

**Figure 6:**
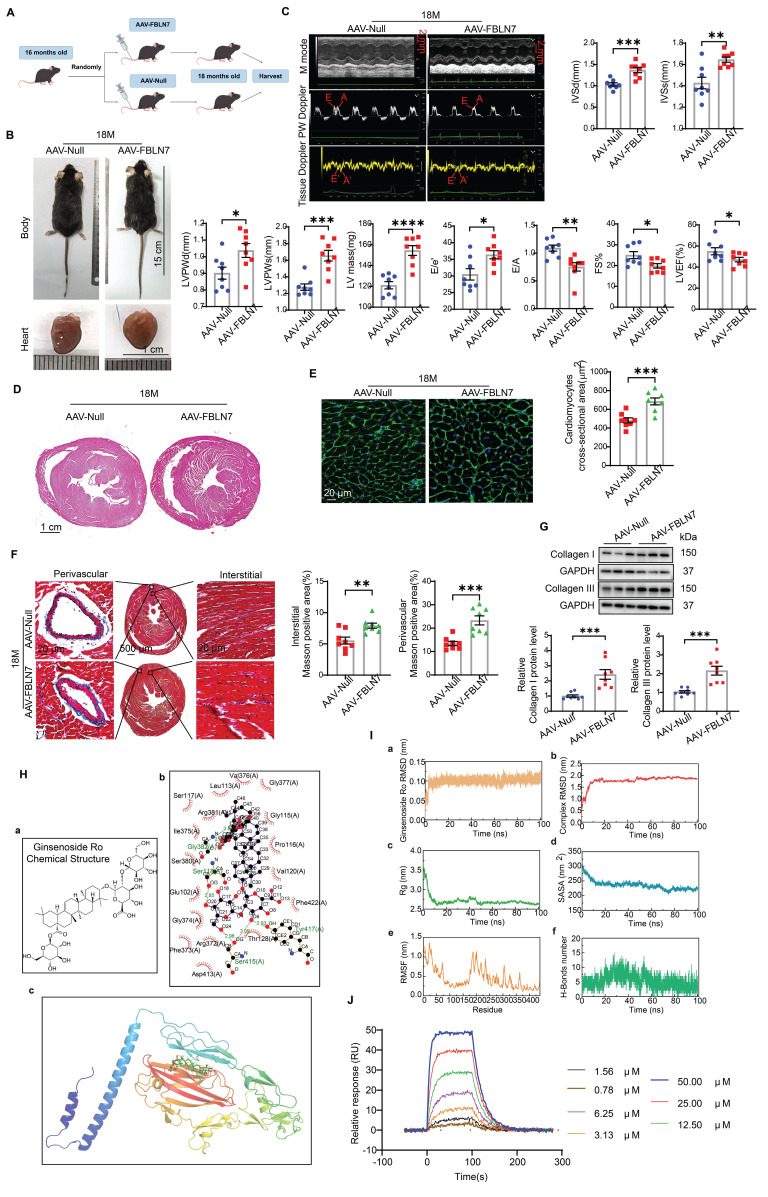
** Overexpression of FBLN7 in FSP1^+^ cells promotes age-related cardiac fibrosis.** A) Schedule for FBLN7 overexpression. B) Gross appearance of mice and hearts in each group. C) Representative echocardiographic images of the 2 groups of mice. Upper: B-mode; middle: PW Doppler mode; lower: tissue Doppler mode. Echocardiographic analysis of interventricular septal thickness in diastole (IVSd) and systole (IVSs), left ventricular posterior wall thickness in diastole (LVPWd) and systole (LVPWs), left ventricular mass, the ratio of early to late diastolic filling velocities (E/A), and the ratio of early diastolic filling velocity to left atrial pressure (E/e'), fractional shortening (FS%) and left ventricular ejection fraction (LVEF) are presented on the right and bottom. D) Representative images of H&E staining of hearts from aging mice (18 months old) injected with AAV-FBLN7 or AAV-Null. E) Representative WGA staining images of the two groups of mice, with quantification shown on the right. F) Representative micrographs of Masson staining in heart sections from AAV-FBLN7 and AAV-Null mice, displaying three different views: upper: perivascular; middle: global; lower: interstitial. Quantifications of fibrotic areas are shown on the right. G) Representative western blot images showing Collagen I and III protein levels in heart tissues from the AAV-Null and AAV-FBLN7 groups. Quantifications are presented on the bottom. H) Results of molecular docking between FBLN7 and Ginsenoside Ro (GRO). a) Structure of GRO. b) Docking zoom map illustrating key amino acid residues at the binding interface and their interactions. c) Predicted structure model of the FBLN7-GRO complex from the GRAMM program, with FBLN7 represented in cartoon form and GRO depicted as sticks. I) Molecular dynamics simulation of the FBLN7-GRO complex over 100 ns. a) Complex root mean square deviation (RMSD) analysis. b) Ligand RMSD analysis. c) Radius of gyration (Rg) analysis. d) Solvent-accessible surface area (SASA) analysis. e) Root mean square fluctuation (RMSF) analysis. f) Hydrogen bond analysis. J) Surface plasmon resonance (SPR) analysis of FBLN7 binding to GRO. Error bars represent the standard error of the mean (SEM). Statistical significance is indicated as follows: *P < 0.05, **P < 0.01, ***P < 0.001, ****P < 0.0001.

To further validate the promotional effect of FBLN7 on age-related cardiac fibrosis via senescent CFs, we overexpressed FBLN7 in mouse fibroblast-specific protein 1 positive (FSP1^+^) cells through tail vein injection using AAV-FBLN7 (Figure 6A). The overexpression of FBLN7 in the heart was confirmed by WB (Figure S6A). The localization of the overexpressed FBLN7 was assessed using IF staining. Confocal images demonstrated the colocalization of overexpressed FBLN7 (Flag-tag) and the CF marker vimentin in the heart (Figure S6B). The gross appearances of the AAV-Null and AAV-FBLN7 mice at 18 months of age are depicted in Figure 6B. The explanted hearts from AAV-Null-18M mice appeared smaller compared to those from AAV-FBLN7-18M mice (Figure 6B). Transthoracic echocardiographic analysis indicated that, when compared to the control group (AAV-Null-18M), the LV internal diameter (LVIDd and LVIDs) did not differ significantly in either AAV-FBLN7-18M group (Figure S6C). However, mice with FSP1^+^ cells overexpressing FBLN7 exhibited greater wall thickness (IVSd, IVSs, LVPWDd and LVPWs), increased LV mass, higher E/e' ratios, and lower E/A ratios, as well as reduced FS% and LVEF (Figure 6C). These findings suggest that FBLN7 overexpression in CFs exacerbates age-related LV dysfunction and hypertrophy. HE and WGA staining corroborated these findings (Figures 6D and 6E). Masson's trichrome staining revealed increased collagen deposition in the interstitial space and perivascular regions of the hearts from AAV-FBLN7-18M mice compared to AAV-Null-18M mice (Figure 6F). Polarized light microscopy of Picro-Sirius Red-stained heart sections demonstrated increases in the areas of Col I and Col III in the interstitial space and perivascular regions following FBLN7 overexpression (Figure S6D). Protein extracted from heart tissue samples was analyzed using WB, and consistent results were obtained (Figure 6G). These data document that the overexpression of FBLN7 in FSP1^+^ cells of mice has pro-hypertrophic and pro-fibrotic effects during aging.

To eliminate the potential indirect effects of blood pressure, body weight, metabolism, cardiac vessel density, and vascular and renal fibrosis on cardiac fibrosis, we examined the impact of FBLN7 overexpression in FSP1^+^ cells on these variables. The results showed that, compared to the AAV-Null group, there were no significant changes in body weight or blood pressure in the AAV-FBLN7 group (Figure S7A and B). Furthermore, there were no notable differences in blood glucose (BG), serum high-density lipoprotein cholesterol (HDL-C), serum low-density lipoprotein cholesterol (LDL-C), serum triglycerides (TG), or serum total cholesterol (TC) levels (Figure S7C-G). Masson staining of the aorta and kidneys indicated no statistically significant difference in the degree of fibrosis between the two groups (Figure S7H and I). Additionally, CD31 immunohistochemical staining of cardiac tissues revealed no significant difference in cardiac vascular density between the AAV-Null and AAV-FBLN7 groups of mice (Figure S7J). Therefore, the potential indirect effects of these factors were excluded.

### Ginsenoside Ro physically interacts with the FBLN7 protein

Natural products have long been recognized as a valuable source for drug discovery. To identify natural products that can inhibit FBLN7 and age-related cardiac fibrosis, a virtual screening analysis was conducted using a natural product database comprising approximately 2,490 compounds. Among these, docking Ginsenoside Ro (GRO) into the binding pocket of FBLN7 yielded the highest docking score (Figure 6H). The stability of the FBLN7-GRO complex was further confirmed through molecular dynamics (MD) simulations (Figure 6I). Based on the root mean square deviation (RMSD) (Figure 6I panel a and b), radius of gyration (Rg) (Figure 6I panel c), and solvent-accessible surface area (SASA) (Figure 6I panel d) data, the system demonstrated stability throughout the simulation, indicating that the binding mode is reliable. The root mean square fluctuation (RMSF) (Figure 6I panel e) and the number of hydrogen bonds (Figure 6I panel f) further support the dynamic behavior of the residues and the binding stability. Additionally, a surface plasmon resonance (SPR) assay was performed to further verify the interaction between FBLN7 and GRO. The results indicated that FBLN7 immobilized on a CM5 chip can bind Ginsenoside Ro with an affinity constant of 1.49 × 10^-5^ M, demonstrating a strong binding affinity of GRO for FBLN7 (Figure 6J). Therefore, it is possible that GRO may exert antifibrotic effects by physically associating with FBLN7.

### GRO restored the impaired profibrotic phenotypes of senescent CFs reversed by FBLN7 overexpression

**Figure 7:**
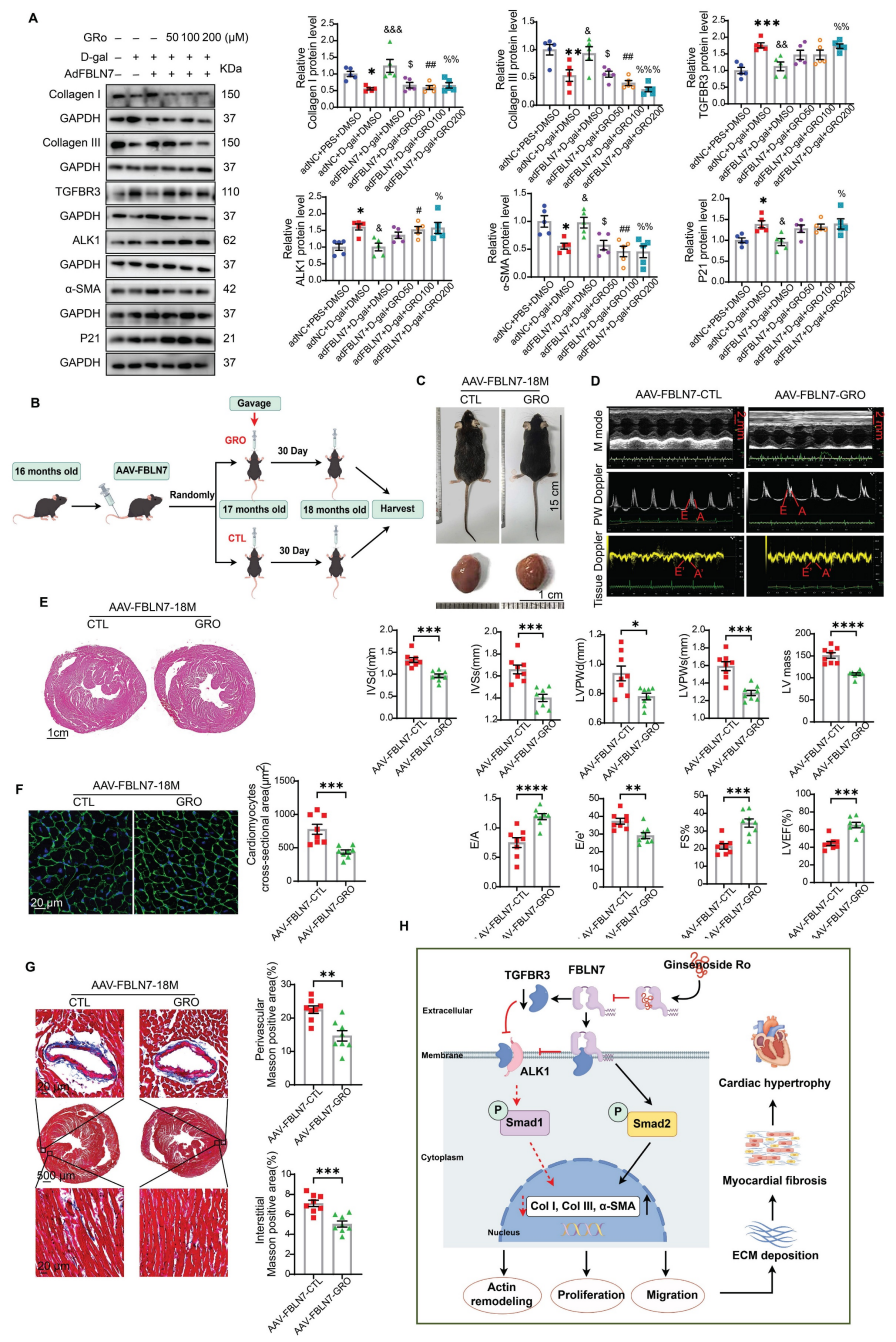
** GRO rescues the impaired profibrotic phenotypes of senescent cardiac fibroblasts and prevents age-related cardiac fibrosis and diastolic dysfunction.** A) Representative Western blot images displaying protein levels of Collagen I and III, TGFBR3, ALK1, α-SMA, and P21 in senescent CFs infected with adenovirus encoding FBLN7 (adFBLN7) and treated with DMSO or various concentrations of GRO (50, 100, and 200 μM). Quantifications are presented on the right. *: adNC+D-gal+DMSO *vs.* adNC+PBS+DMSO, &: adFBLN7+D-gal+DMSO *vs*. adNC+D-gal+DMSO, $: adFBLN7+D-gal+GRO50 *vs.* adFBLN7+D-gal+DMSO, #: adFBLN7+D-gal+GRO100 *vs.* adFBLN7+D-gal+DMSO, %: adFBLN7+D-gal+GRO200 *vs.* adFBLN7+D-gal+DMSO. B) Schedule for FBLN7 overexpression and GRO supplementation in mice. C) Gross appearances of mice and their hearts in each group. D) Representative echocardiography images of the two groups of mice. Upper: B-mode; middle: PW Doppler mode; lower: tissue Doppler mode. Echocardiographic analysis of interventricular septal thickness in diastole (IVSd) and systole (IVSs), left ventricular posterior wall thickness in diastole (LVPWd) and systole (LVPWs), left ventricular mass, the ratio of early to late diastolic filling velocities (E/A), and the ratio of early diastolic filling velocity to left atrial pressure (E/e'), left ventricular ejection fraction (LVEF) and fractional shortening (FS%) are shown at the bottom. E) Representative images of H&E staining of hearts from aging mice (18 months old) injected with AAV-FBLN7 and administered 40 mg/kg/d of GRO or solvent control via gavage. F) Representative WGA staining images of the two groups of mice, with quantification shown on the right. G) Representative micrographs of Masson staining in heart sections from the AAV-FBLN7-18M-GRO and AAV-FBLN7-18M-CTL mice, displaying three different views: upper: perivascular; middle: global; lower: interstitial. Quantifications of fibrotic areas are presented on the right. H) FBLN7 exerts its reversing effect on the impaired profibrotic phenotypes of senescent CFs by downregulating the TGFBR3/ALK1/Smad1 pathway, which may contribute to the pathogenesis of age-related cardiac fibrosis. GRO exerts antifibrotic effects possibly through interaction with FBLN7. Error bars represent the standard error of the mean (SEM). Statistical significance is indicated as follows: *P < 0.05, **P < 0.01, ***P < 0.001, ****P < 0.0001.

Having made this observation, we next verified whether GRO could restored the impaired profibrotic phenotypes of senescent CFs that are reversed by FBLN7 overexpression. The selection of drug concentrations was based on published literature 21, 22. As anticipated, GRO treatment restored the phenotypes of senescent CFs that had been reversed by FBLN7 overexpression, as indicated by reduced proliferation and migration capacities (Figure S8A and B), decreased levels of Col I and Col III proteins (Figure 7A), and impaired expression of α-SMA (Figure 7A and Figure S8D) in FBLN7-overexpressing senescent CFs following GRO treatment (adFBLN7-D-gal-GRO) compared to FBLN7 overexpression alone (adFBLN7-D-gal-CTL). As shown in WB and SA β-gal staining, the P21 protein level (Figure 7A) and the number of senescent CFs (Figure S8C) increased after GRO exposure followed by FBLN7 overexpression, compared to senescent CFs with only FBLN7 overexpression. Additionally, GRO treatment rescued the decreases in TGFBR3 and AK1 protein levels induced by FBLN7 overexpression, suggesting that GRO likely acts by interfering with FBLN7 function (Figure 7A). Furthermore, we observed that indicators representing different phenotypes, or detected by various methods, responded differently to the same concentration of GRO. However, at a GRO concentration of 200 μM, all indices demonstrated statistically significant differences between the adFBLN7-D-gal-GRO and adFBLN7-D-gal-CTL groups. We also investigated the effect of GRO on the expression of FBLN7. The results indicated that a GRO concentration of 200 μM significantly reduced the expression of FBLN7 (Figure S8E). These results indicate that GRO treatment in FBLN7-overexpressing senescent CFs represses proliferation and migration and alleviates collagen synthesis, likely by inhibiting FBLN7-mediated downregulation of TGFBR3.

### GRO prevented age-related cardiac fibrosis and dysfunction exacerbated by the overexpression of FBLN7

We further confirmed the cardioprotective function of GRO using mice with FSP1^+^ cells overexpression of FBLN7. The timing of overexpression and GRO supplementation is illustrated in Figure 7B. Gross images of the control group (AAV-FBLN7-CTL) and the GRO treatment group (AAV-FBLN7-GRO) are presented in Figure 7C. The explanted hearts from AAV-FBLN7-GRO mice appeared smaller compared to those from AAV-FBLN7-CTL mice. Transthoracic echocardiographic analysis revealed that mice with FSP1^+^ cells overexpressing FBLN7 treated with GRO exhibited a larger LV internal diameter (LVIDd and LVIDs) (Figure S9A), reduced wall thickness (IVSd, IVSs, LVPWd and LVPWs), decreased LV mass, increased E/A ratios, and lower E/e' ratios. These findings indicate that GRO treatment mitigated age-related LV diastolic dysfunction and hypertrophy exacerbated by FBLN7 overexpression (Figure 7D). When compared to the AAV-FBLN7-CTL group, the LVEF and FS% were elevated in the AAV-FBLN7-GRO group, indicating that GRO treatment also improved the cardiac systolic function affected by FBLN7 overexpression (Figure 7D). HE and WGA staining displayed similar results (Figures 7E and F). Masson's trichrome staining demonstrated reduced collagen deposition in the interstitial space and perivascular region of the hearts from AAV-FBLN7-GRO mice compared to those from AAV-FBLN7-CTL mice (Figure 7G). WB analysis also detected a decrease in the protein levels of Col I and Col III in the myocardial tissues of mice in the GRO treatment group (Figure S9B). These data indicate that GRO treatment can attenuate age-related cardiac fibrosis and improve cardiac dysfunction exacerbated by the overexpression of FBLN7 in FSP1^+^ cells.

## Discussion

In our study, we elucidated the role of FBLN7 as a significant profibrotic factor in the aging heart. Our results demonstrated the cardioprotective effects of FBLN7 KO in aged mice, which reduces age-related myocardial fibrosis and ultimately enhances cardiac diastolic function. The observation that overexpressing FBLN7 in FSP1^+^ cells of aged mice exacerbates age-related myocardial fibrosis reinforces this finding. Mechanistically, we identified that FBLN7 promotes the proliferation, migration, actin remodeling, and collagen production of senescent CFs partially by inhibiting the TGFBR3/ALK1/Smad1 pathway. Additionally, we identified a natural product, GRO, that physically interacts with FBLN7 and validated its antifibrotic activity both in vitro and in vivo. Given that age-related fibrosis is a critical pathological factor in HFpEF, downregulating FBLN7 and/or interfering with its function may represent an effective therapeutic strategy for HFpEF.

FBLN7 is an extracellular matrix protein that modulates cell adhesion, migration, proliferation, and differentiation 10. We have previously demonstrated that FBLN7 promotes myocardial fibrosis through the activation of CFs under pathological conditions 11. A recent study indicated that FBLN7 contributes maintaining tissue resilience and the heterogeneity of epidermal stem cells during skin aging 23. However, the function of FBLN7 in myocardial fibrosis within the context of aging remains unclear. In our study, we confirmed that FBLN7 accumulates in aging hearts and validated its profibrotic function by constructing FBLN7 KO and overexpression models in naturally aging mice. These findings are consistent with previous studies regarding the effects of Fibulins on fibrosis, including our earlier work 11, 12, 24-26.

Since FBLN7 global KO mice were used in the present study, observed changes in protective effects of FBLN7 KO may reflect effects of FBLN7 on other tissues and/or pathophysiological progress. Compared to the WT-18M group, we found that FBLN7-KO-18M group shows lower total cholesterol, low-density lipoprotein cholesterol, triglycerides and higher blood glucose (Figure S10). The rearrangement and accumulation of lipids are associated with cardiac fibrosis 27. In addition, multiple models of hypertrophy show an increased rate of glucose uptake and glycolysis 28, 29, which is consistent with increased reliance on glucose transporter 1 (GLUT1)-dependent glucose transport and a switch to a fetal metabolic profile 30. The protective effects of FBLN7 KO in ageing hearts may associated with increasing myocardial glycogen and/or improving 'energy reserve', as the impaired myocardial energetics occurs in cardiac hypertrophy. Future studies could investigate the mechanisms linking FBLN7 and metabolic abnormalities.

Notably, we found that the deletion of FBLN7 alleviates age-related fibrosis possibly by promoting CF senescence. As age increases, CFs also exhibit aging phenotypes, such as impaired proliferation, migration, and collagen production capacities. Our results confirm that FBLN7 can suppress fibroblast senescence. There is still controversy surrounding the role of fibroblast senescence in heart diseases 31. However, recent studies suggest that fibroblast senescence may help limit diffuse myocardial fibrosis 32, 33. Our research also demonstrates that the impaired profibrotic phenotypes of senescent fibroblasts are beneficial in alleviating myocardial fibrosis, indicating that the induction of fibroblast senescence could be a viable strategy to inhibit tissue fibrosis.

One interesting finding in this study is the significant increase in FBLN7 protein levels in aging hearts, while a decrease is observed in senescent CFs. IF staining revealed that, in addition to CFs and MCFs, FBLN7 also co-localizes with other cell types, such as endothelial cells (ECs) and smooth muscle cells (SMCs), in aging hearts. Given that FBLN7 is a member of the extracellular matrix proteins produced by nearly all cell types in the heart, it is likely that these other cell types contribute to the accumulation of FBLN7 34. Senescent CFs exhibit reduced proliferative and collagen synthetic capacities 35. Current studies suggest that CF senescence may be beneficial in mitigating age-related cardiac fibrosis, indicating that inducing CF senescence could be a viable strategy to inhibit cardiac fibrosis 32, 33. Consistently, our study confirmed that the upregulation of FBLN7 functions by inhibiting aging phenotypes and promoting profibrotic phenotypes in senescent CFs. Therefore, it is reasonable to conclude that FBLN7, as a pro-fibrotic factor in myocardial fibrosis, is elevated in aging hearts and diminished in impaired profibrotic senescent CFs. Although we have established the role of FBLN7 in senescent CFs contributing to its profibrotic effects both in vivo and in vitro, the source and regulation of FBLN7, as well as its role in intercellular communication, remain uncertain.

ALK1 is a serine-threonine kinase receptor that belongs to the TGF-β receptor I (TGFBR1) family. It is phosphorylated by ligand-bound TGFBR2, which activates Smad1/5 36, 37. The role of the ALK1-Smad1/5/9 pathway in fibrotic disorders remains controversial, as it appears to play different, and sometimes opposing, roles depending on the cell type and context 38. In a mouse model of obstructive nephropathy, heterozygous disruption of ALK1 promoted renal fibrosis at 15 days following unilateral ureteral obstruction, but mitigated renal fibrosis at 3 days of obstruction 39, 40. In various cell types, including ECs, renal fibroblasts, skin fibroblasts, and human chondrocytes, ALK1 appears to counteract TGF-β1/ALK5-induced ECM protein expression 39, 41-44. Conversely, it has been reported that the ALK1-Smad1/5 pathway promotes a fibrotic phenotype in scleroderma fibroblasts 45. TGF-β1 facilitates the transdifferentiation of hepatic stellate cells (HSCs) into myofibroblasts, contributing to liver fibrosis through the ALK1-Smad1/5 pathway 46. In the context of myocardial fibrosis, current studies suggest that ALK1 plays a cardioprotective role. In a murine model of transverse aortic constriction (TAC)-induced heart failure, reduced expression of ALK1 was associated with increased cardiac fibrosis and impaired LV function 18. Following myocardial infarction (MI), ALK1 deficiency was found to increase post-MI mortality and promote cardiac remodeling 47. Consistently, we observed that the ALK1-Smad1/5/9 pathway is upregulated in senescent CFs and plays an anti-fibrotic role in FBLN7-mediated collagen deposition. Previous studies have suggested that the ALK1 cascade antagonizes ALK5 signaling responses 48. In this study, the anti-fibrotic function of the ALK1-Smad1/5/9 pathway may not be solely attributed to the antagonism of ALK5/Smad2 signaling, as inhibition of ALK1 abolished the anti-fibrotic effect of FBLN7 knockdown without affecting ALK5/Smad2 activation. However, the specific mechanism by which ALK1 exerts its anti-fibrotic effects independently of ALK5 remains unclear and requires further investigation.

The TGFBR3 is the most abundantly expressed TGF-β co-receptor known to bind all three isoforms of TGF-β and regulate TGF-β signaling 49. Growing evidence suggests that upregulating TGFBR3 expression may be a promising strategy for the prevention or treatment of ECM deposition 19, 20, 50-52. Consistent with these findings, we observed that the overexpression of TGFBR3 can reduce collagen synthesis induced by FBLN7 overexpression in senescent CFs. The anti-fibrotic effect of TGFBR3 is currently believed to be mediated by antagonizing ALK5-Smad2/3 signaling. In the context of hepatic fibrosis, decreased β-arrestin2 has been shown to inhibit HSCs collagen production and TGF-β1-Smad2/3 pathway activation by elevating TGFBR3 expression 52. Our results indicate that TGFBR3 differentially regulates TGF-β signal transduction by inhibiting the ALK5-Smad2 pathway while augmenting the ALK1-Smad1/5/9 pathway in senescent CFs. Furthermore, TGFBR3 regulates the internalization of ALK5 and TGFBR2, but not ALK1, which may elucidate the mechanism by which TGFBR3 modulates the switching between these two distinct TGF-β signaling pathways 53.

In addition, we observed that FBLN7 not only binds to TGFBR3 but also reduces its protein levels. However, whether the effect of FBLN7 on TGF-β signal transduction results from its binding to TGFBR3, a reduction in TGFBR3 expression, or a combination of both has yet to be determined. It is possible that the FBLN7-TGFBR3 interaction interferes with the presentation of TGFBR3-mediated ligands, thereby altering the activation of downstream signaling pathways 54. We have previously demonstrated that FBLN7 regulates actin cytoskeletal reorganization, which is crucial for receptor endocytosis, transport, and degradation or recycling 12, 55-57. Therefore, another possibility is that FBLN7 binds to TGFBR3 and modifies actin cytoskeletal reorganization, leading to defective externalization or internalization of TGFBR3 and a subsequent reduction in TGFBR3 levels. Given the potential role of the FBLN7-TGFBR3 interaction in other age-related diseases, such as tumors and osteoarthritis, these mechanisms warrant further investigation.

As an emerging therapeutic agent, ginsenosides exhibit significant potential in the prevention and treatment of obesity, diabetes, cardiovascular diseases, and related conditions 58. Several ginsenosides have been shown to alleviate cardiac fibrosis 59-61; however, the role of GRO in age-related cardiac fibrosis remains unclear. Our research identified several bioactive compounds that potentially bind to FBLN7 through virtual screening, and GRO was determined to stably bind to FBLN7 by MD simulation and SPR. Furthermore, we demonstrated that GRO intervention inhibited the profibrotic phenotypes promoted by FBLN7 overexpression in aging CFs, as well as diastolic dysfunction and myocardial fibrosis in aging mice. Our findings position GRO as a promising therapeutic target for age-related cardiac fibrosis, likely by inhibiting the action of FBLN7. Additionally, we found that with the supplementation of GRO, the protein level of FBLN7 decreased. Therefore, GRO may also improve age-related myocardial fibrosis by reducing the level of FBLN7. These results further support the notion that ginsenosides play a protective role in cardiac fibrosis 62-65. Moreover, we observed that the antifibrotic effect of GRO on cardiac fibrosis surpasses the profibrotic effect of FBLN7 overexpression. It is plausible that GRO may act through other targets in CFs and/or other cell types. A recent study demonstrated that GRO prevents endothelial injury via promoting Epac1/AMPK-mediated mitochondrial protection in early diabetic retinopathy 66. GRO also exerts anti-inflammatory actions through the direct inhibition of the TLR4 signaling pathway 67. Since mitochondrial protection and anti-inflammatory effects play a significant role in preventing myocardial fibrosis, GRO may improve age-related myocardial fibrosis through these mechanisms, necessitating further research to identify additional potential targets and mechanisms of action for GRO.

This study, however, has several limitations. Although FBLN7 is a secreted protein, we did not confirm the source of FBLN7 accumulation in aging hearts, despite observing that FBLN7 primarily co-localized with CFs and MCFs. Furthermore, our focus was primarily on the role of FBLN7 in the profibrotic and aging phenotypes of CFs. Myocardial fibrosis is indeed a complex and multifactorial process in the context of aging, involving distinct phenotypes of multiple cell types. Other phenotypes of CFs mediated by FBLN7 may also be implicated, such as the pro-inflammatory phenotype. Finally, due to the gender differences in cardiovascular diseases, the animal phenotypes in this study may be limited to males, as male mice were used in this study.

Taking all factors into account, our results demonstrate that FBLN7 is a key regulator of myocardial fibrosis in the context of aging and reveals its potential to reverse the impaired profibrotic phenotypes of senescent CFs, which may contribute to the pathogenesis of age-related cardiac fibrosis. Future therapeutic strategies that directly inhibit FBLN7 could represent an effective approach to limit cardiac fibrosis and its associated disorders.

## Methods

### Animals

The generation of the FBLN7-KO was commissioned by Cyagen Biosciences Inc (Guangzhou, China). To generate knockout mice, the CRISPR/Cas9 strategy was used to delete exons 2-3 of mouse Fbln7 via non-homologous recombination. FBLN7-KO mice were then obtained by mating the FBLN7 heterozygous mice. Subsequent genotyping was performed by PCR using the genomic DNA obtained from clipped tails with the following primers: forward 5'-AAGACATAAACATCAACCTCTGGC-3' and reverse 5'-CACATTGCTCTTGCATTTGTGTG-3.'

The adeno-associated virus serotype 9 (AAV9) vector, driven by the fibroblast-specific protein-1 (FSP1) promoter and containing the gene sequence for Fbln7 (designated as AAV-FBLN7), was utilized for in vivo overexpression studies. An AAV9-empty vector (AAV-NC) served as the control (WZ Biosciences Inc., Shandong, China). Mice were randomly injected with either AAV-FBLN7 or AAV-NC via the tail vein at a dosage of 5 × 10^11^ vector genomes (vg) per mouse.

### Animal model

The 18-month-old male C57BL/6J mice were utilized as a natural aging model to simulate cardiac fibrosis, ventricular remodeling, and diastolic dysfunction. FBLN7 knockout (KO) mice and wild-type (WT) littermate controls were reared concurrently under specific-pathogen-free conditions.

Wild-type C57BL/6 mice from Zhishan Healthcare Research Institute Ltd were utilized at 15 months of age for experiments involving FBLN7 overexpression. Following the injection, the mice were housed under specific-pathogen-free conditions until they reached 18 months of age.

FBLN7-overexpressing mice were gavaged daily with 40 mg/kg of Ginsenoside Ro (GRO) (HY-N0607, MCE) or a solvent control (10% DMSO+40% PEG300+5% Tween-80+45% Saline) at 17 months of age for one month.

At least eight mice were included in each group for every experiment. All mouse experiments were conducted in accordance with the general guidelines established by the Association for Assessment and Accreditation of Laboratory Animal Care, and were approved by the Laboratory Animal Committee of Shandong University Qilu Hospital (Jinan, Shandong Province, China) (Permit number: DWLL-2023-089).

A D-galactose-induced mouse aging model was established through subcutaneous injection of D-galactose (150 mg/kg/day, Sigma-Aldrich Corporation, St. Louis, MO, USA) or 0.9% saline (control) for a duration of three months.

### Western blot

Cells and heart tissues were collected, and lysates were prepared. An equal quantity of protein (40-60 μg) was resolved by SDS/PAGE and transferred to polyvinylidene difluoride membranes. The blots were blocked with 5% milk in Tris-buffered saline with Tween 20 for 2 h at room temperature (RT) and then separately incubated with primary antibodies overnight at 4 °C. The blots of p-Smad2 and p-Smad1/5/9 were blocked with Rapid Blocking Buffer (RM02956, ABclonal Biotechnology Co., Ltd.) for 15 min at RT and incubated with primary antibodies for 24 h at 4 °C. The blots were then incubated with secondary antibodies conjugated to horseradish peroxidase for 1 h at RT and detected using a chemiluminescent instrument (GE, Amersham Imager 680 RGB and 600 RGB). Grayscale values for each blot were measured using the ImageJ software (National Institutes of Health, Bethesda, MD, USA), and the intensity of each band was normalized to that of the loading control GAPDH or the total target protein. Detailed information regarding the antibodies used is provided in Table S1 (Supporting Information).

### Enzyme-linked immunosorbent assay

FBLN7 levels in serum and culture supernatants were determined using enzyme-linked immunosorbent assay (ELISA) kits following the manufacturer's instructions (Mmbio, Jiangsu, China and Cloud-Clone Corp, Wuhan, China).

### Echocardiography

Transthoracic echocardiography was conducted using a VisualSonics Vevo 3100 or 2100 system equipped with an MS-400 linear transducer to assess parameters of ventricular remodeling and cardiac function. The measured parameters included interventricular septal thickness in diastole (IVSd), interventricular septal thickness in systole (IVSs), left ventricular internal diameter in diastole (LVIDd), left ventricular internal diameter in systole (LVIDs), left ventricular posterior wall thickness in diastole (LVPWd), left ventricular posterior wall thickness in systole (LVPWs), left ventricular mass, left ventricular ejection fraction (LVEF), fractional shortening (FS%), the ratio of early to late diastolic filling velocities (E/A), and the ratio of early diastolic filling velocity to left atrial pressure (E/e'). This procedure was performed at the conclusion of the experiments, during which five consecutive cardiac cycles were analyzed, and the results were recorded.

### Histopathological staining

Isolated heart tissues were immediately fixed in a 4% paraformaldehyde solution, followed by routine dehydration, paraffin embedding, and serial sectioning (4 μm).

• H&E staining was conducted according to standard protocols.

• Masson's staining was performed using a modified Masson's trichrome staining kit (G1346, Solarbio, Beijing, China,).

• Picrosirius red staining was performed by staining the heart sections in a freshly prepared 0.1% picrosirius red solution for 2 h at room temperature in the dark.

• IHC staining: the heart sections were deparaffinized in xylene and rehydrated in graded ethanol. Antigen retrieval was performed according to the methods recommended in the Antibody Manual. Endogenous peroxidase activity was quenched with 3% H_2_O_2_ for 15 min, and sections were blocked in 5% goat serum for 30 min. Incubations with primary antibodies were performed overnight at 4 °C in a humidified chamber, followed by appropriate HRP-conjugated secondary antibodies for 1 h at room temperature. A DAB Kit (ZSGB-Bio, Beijing, China) was used to develop signals according to the manufacturer's instructions. Finally, the sections were counterstained with hematoxylin.

• Immunofluorescence (IF) staining of heart slides was performed similarly to IHC staining, except that the sections were blocked in 5% goat serum after antigen retrieval and subsequently incubated with primary antibodies overnight. The next day, the slides were incubated with Alexa Fluor conjugated secondary antibodies for 1 h at room temperature and mounted using a mounting medium containing DAPI (Abcam, ab104139).

The antibodies used are detailed in Table S1 (Supporting Information). Confocal images were captured using an Andor spinning disk confocal microscope (Andor, Oxford Instruments). Other stained sections were visualized and photographed using the Pannoramic Scanner with Pannoramic Viewer Software (3D HISTECH, Budapest, Hungary) and analyzed with ImageJ software (National Institutes of Health, Bethesda, MD, USA).

### Cardiac fibroblast isolation and culture

Cardiac fibroblasts (CFs) were isolated from neonatal mice, as previously described. Neonatal mouse CFs were cultured in DMEM (Basal Media, Shanghai, China) with 10% fetal bovine serum (FBS) (Cellbox, Australia) and 1% penicillin/streptomycin at 37 °C under 5% CO_2_ in a humidified incubator.

### Cardiac fibroblast treatment

Primary CFs were treated with recombinant human FBLN7 protein (8530-FB, RD Systems) at various concentrations (negative control, 100, 200, 400, 800 and 1600ng/mL) to investigate the direct effect of FBLN7 on CFs.

Senescent CFs were induced by treatment with 20 mg/mL D-gal (G0750, Sigma-Aldrich) for 48 h. Phosphate-buffered saline (PBS)-treated or early-passage CFs (passages 3-6) were used as non-senescent controls.

When indicated, CFs were pretreated with 250 nM ALK1 inhibitor (ML347, MCE, Shanghai, China) or 0.01% DMSO (Sigma-Aldrich) for 2 h prior to a 48-hour treatment with D-gal.

### Bioinformatic analysis

Extract the proteins from left ventricular tissues of wild-type and FBLN7-KO aged mice. Appropriate number of peptides were taken from each sample, and chromatographic separation was performed using Vanquish Neo UHPLC system and operated Neo UHPLC chromatographic system (Scientific). DIA raw data files were analyzed by DIA-NN software (v 1.9). A spectral library was obtained by the deep learning algorithm in DIA-NN, and the DIA raw data were extracted with the obtained spectral library as well as the spectral library obtained by the MBR function to obtain protein quantification information. The final results were screened at 1% FDR for parent ions and protein levels. The quantitative proteomic information after screening was used for subsequent analyses. (1) GO annotation of differential proteins. DIA raw data files were analyzed by DIA-NN software (v 1.9). A spectral library was obtained by the deep learning algorithm in DIA-NN, and the DIA raw data were extracted with the obtained spectral library as well as the spectral library obtained by the MBR function to obtain protein quantification information. The final results were screened at 1% FDR for parent ions and protein levels. The quantitative proteomic information after screening was used for subsequent analyses. (2) KEGG annotation of differential proteins. Following annotation steps, the studied proteins were blasted against the online Kyoto Encyclopedia of Genes and Genomes (KEGG) database (http://geneontology.org/) to retrieve their KEGG ortholog identifications and were subsequently mapped to pathways in KEGG.

### Constructions of plasmid, siRNA, and adenovirus

Full-length cDNA of mouse TGFBR3 and FBLN7 were amplified by standard PCR and subcloned into the pcDNA 3.1 vector with a C-terminal Flag-tag as well as a C-terminal EGFP-tag and C-terminal Myc-tag. The pcDNA 3.1-Flag, pcDNA 3.1-EGFP, and pcDNA 3.1-Myc plasmids were used as controls. Small interfering RNA (siRNA) oligonucleotides against FBLN7 (siFBLN7) and negative control siRNA (siNC) were synthesized by GenePharma (Shanghai, China). The sequence of siFBLN7: 5′-CGUGGUGUGUCUUGCUAAUTT-3′; siNC: 5′-UUCUCCGAACGUGUCACGUTT-3′. siRNA oligonucleotides against TGFBR3 (siTGFBR3) and siNC were synthesized by Cloud-Clone Corp (Wuhan, China). The sequence of siFBLN7: 5′-CCACAGAGAGCCAGAAGAATT-3′; siNC: 5′-UUCUCCGAACGUGUCACGUTT-3′. Recombinant adenoviruses encoding FBLN7 (adFBLN7) and an adenovirus vector (adNC served as a negative control) were designed by GeneChem (Shanghai, China).

### Transfection and infection

CFs were transfected with siFBLN7 and siNC using Lipofectamine RNAiMAX transfection reagent (Invitrogen, USA) to knock down FBLN7 in vitro, according to the manufacturer's protocol. For adenovirus-mediated in vitro overexpression, CFs were incubated with adFBLN7 and adNC for 6-8 h at a multiplicity of infection (MOI) of 50 PFU per cell. All constructed plasmids were transiently transfected using Lipofectamine 3000 (Invitrogen) according to the manufacturer's instructions.

### Immunocytochemistry (confocal microscopy)

The cells were fixed in 4% paraformaldehyde for 15 min, permeabilized with 0.1% Triton-X100 in PBS for 10 min, and blocked in 5% goat serum for 0.5 h at room temperature. Next, the cells were incubated with primary antibodies or PBS (served as a negative control) overnight at 4 °C. Appropriate Alexa Fluor-conjugated secondary antibodies were used for fluorescence staining. Nuclei were stained with DAPI (Abcam, ab104139). Confocal images were acquired using an Andor spinning disk confocal microscope (Andor, Oxford Instruments). Other fluorescent immunocytochemical images were captured using a fluorescence microscope (Carl Zeiss).

### Transwell migration assay

The 24-well Transwell plate (8 μm pore size, Corning, Lowell, MA, USA) was utilized to conduct Cell migration experiments. CFs (approximately 5 × 10^4^) were mixed with 200 μL serum-free medium and added into the upper chamber of the insert. Then, 800 μL 20% FBS culture medium was added to the bottom chamber and incubated for 24 h. The non-migratory cells on the upper side of the chamber membrane were removed using cotton swabs, while migratory cells on the bottom surface were fixed in cold methanol and stained with Crystal violet (0.1%). Finally, cells were imaged with microscopes and the number of migrating cells in three random fields of each sample was counted using Image J software.

### Senescence-associated β-gal staining

Senescence-associated β-gal staining performed to assess the senescence state of the CFs using the Cell Senescence β-galactosidase Staining Kit (C0602, Beyotime, Shanghai, China), according to the manufacturer's instruction. Images were captured using a Zeiss fluorescence microscope. The percentage of positive cells was analyzed using ImageJ software 2.0 (National Institutes of Health, Bethesda, MD, USA).

### 5-Ethynyl-2′-Deoxyuridine Assay

The cell proliferation was detected by 5-Ethynyl-2′-Deoxyuridine (EdU) assay using a Cell-light EdU Apollo567 In Vitro Kit (C10310-1, RiboBio, Guangzhou, China), in accordance with the manufacturer's protocol. Images were captured using a Zeiss fluorescence microscope. The percentage of positive cells was analyzed using ImageJ software 2.0 (National Institutes of Health, Bethesda, MD, USA).

### Co-Immunoprecipitation

Twenty-four hours after plasmid transfection, the cells were harvested and lysed in nondenaturing lysis buffer (P0013, Beyotime Biotechnology. Shanghai, China) for 30 min. Clear lysates were incubated with Immunoprecipitation (IP)-grade antibodies or IgG for 1 h, followed by the addition of pretreated protein A/G magnetic beads (HY K0202; MedChemExpress, Monmouth Junction, NJ, USA) to a rotating wheel at 4 °C overnight. The next day, the magnetic beads were recovered, and the supernatant was discarded. After washing the beads four times with PBS containing 0.5% Tween-20, SDS-PAGE loading buffer was added, and the mixtures were heated to 95°C for 5 min. The collected supernatants were used for western blot analysis.

### Molecular modeling and docking

The crystal structures of FBLN7 (AF-Q501P1-F1-v4) and TGFBR3 (AF-O88393-F1-v4) were obtained from AlphaFold prediction. Rigid protein-protein docking was carried out using the GRAMM-X server (http://gramm.compbio.ku.edu/) to investigate the binding interaction between FNLN7 and TGFBR3. The output docked conformation with the highest score was considered the binding conformation, and the docking results were visualized using PyMol 2.6 (PyMOL Molecular Graphics System).

### Library preparation and virtual screening

About 2,490 natural products (Cat. No.: L1400) were downloaded from the Selleck (https://www.selleck.cn/) database, and the X-ray crystal structure of FBLN7 was downloaded from the AlphaFold Protein Structure Database (https://alphafold.ebi.ac.uk/entry/Q53RD9). The virtual screening was performed using the AutoDock Vina v1.2.3 software, which will automatically search for the optimal binding site and conformation during the run docking process 68, 69. The interpretation of H-bonds between FBLN7 and compounds were visualized through PyMol v2.6 software 70 and Ligplot+ v2.2.8 software 71.

### Molecular dynamics simulation

The molecular docking model of the FBLN7 and GRO was taken as starting conformation for molecular dynamics (MD) simulations. Simulations were carried out with the Gromacs v2022.03 software, the CHARMM36 force field, and the original three-point transferable intermolecular potential (TIP3P) water model. The FBLN7-GRO complex was placed in a dodecahedral box, where the distance between the protein to the box edges was at least 1.2 nm. The simulated system is neutralized by adding appropriate amounts of Na^+^ and Cl^-^ (concentration: 0.154 M). The steepest decent algorithm was used to carry out energy minimization (EM). For short range electrostatic and van der Waals interactions, the cutoff distance is set to 1.0 nm, and for long range electrostatic interactions, the Particle Mesh Ewald (PME) method is used to calculate electrostatic interactions. The LINCS algorithm is used to constrain all involved hydrogen bonds. The pressure was controlled at 1 bar with the Berendsen, and the simulated temperature was adjusted using a V-rescale temperature coupling method. Subsequently, the protein structure is pre-balanced by 100 ps to optimize the initial conformation of the protein in the solvent, balanced 100 ps in an isothermal equal volume (NVT) system to heat the protein and solvent system to the target temperature, as well as balanced 100 ps in an isothermal isobaric (NPT) system, so that the solvent and protein system pressure balance. Finally, a time molecular dynamics simulation of 100 ns was performed and the simulated trajectories were saved for subsequent analysis. Based on the results of the MD simulation, the RMSD (root mean square deviation), RMSF (root mean square fluctuation), Rg (radius of rotation) values, SASA (solution accessible surface area) and the number of statistical hydrogen bonds were calculated. The Gibbs free energy, molecular mechanics/Poisson-Boltzmann surface area (MM/PBSA) and binding free energy of the complex were calculated using Gromacs v2022.03 software.

### Surface plasmon resonance (SPR)

SPR binding experiments were performed using a Biacore apparatus equipped with CM5 sensor chips (GE Healthcare). The FBLN7 recombinant protein was immobilized on CM5 sensor chips using an amine-coupling strategy. The sensor chip was activated with an activation buffer (0.4 M EDC+0.1 M NHS). FBLN7 solution (20 μg/mL, solubilized in 10 mM sodium acetate buffer, pH 4.5) was injected into the sample channel at a flow rate of 10 μL/min to reach a level of immobilization of 12600 relative units (RU). The reference channel did not require a ligand-immobilization step. The chip was blocked by injecting an ethanolamine solution (1 M Ethanolamine hydrochloride). Ginsenoside Ro was dissolved in DMSO and diluted with the analyte buffer (1% DMSO PBST, 0.005% Tween) to 8 concentrations (0.75-50 μM), which were then injected into both the sample and reference channels at a flow rate of 20 μL/min for an association phase lasting 100 s, followed by a dissociation phase of 180 s. All tests were performed at 22 °C. The binding kinetic of Ginsenoside Ro over the FBLN7 sensor chip was evaluated in the analyte buffer. Eight cycles of analyte injection were repeated according to the ascending concentrations of Ginsenoside Ro. After each cycle of interaction analysis, the chip was regenerated by injecting 100 μL regeneration buffer (10 mM Glycine-HCl, pH = 2.0) at a flow rate of 200 μL/min. Binding sensograms were obtained by subtracting the response from the reference flow cell. Data analysis was performed using BIA Evaluation Software (GE Healthcare) and fitted to a one-site Langmuir binding model.

### Statistical Analysis

All results were shown as mean ± SEM unless otherwise indicated. The Kolmogorov-Smirnov test was used to confirm the normal distribution of the data. The unpaired, two-tailed Student's t-test was used for comparison between two groups with normally distributed data, whereas the two-tailed Mann-Whitney U test was used for data that did not comply with a normal distribution. For univariate comparisons between multiple groups, a one-way ANOVA followed by Dunnett's or Tukey's post-hoc tests were used for data with a normal distribution; otherwise, data were analyzed using the Kruskal Wallis test followed by the Dunn post-hoc test. For multivariate comparisons between groups, a two-way ANOVA followed by the Sidak post-hoc test was used. GraphPadPrism8 (GraphPad Software, LaJolla, CA, USA) was used for the statistical analyses. Statistical significance was set at p < 0.05.

## Supplementary Material

Supplementary figures and table.
