# Supplementary material for: FBLN7 KO attenuates age-related cardiac fibrosis by promoting TGFBR3/ALK1/Smad1 signaling and inhibiting the profibrotic phenotypes of cardiac fibroblasts
Source: Theranostics. 2025 Jul 28;15(16):8531–52. doi: 10.7150/thno.116477 (PMC12374634; doi:10.7150/thno.116477)
Supplement: Supplementary file 1 — Supplementary figures and table. [file thnov15p8531s1.pdf]

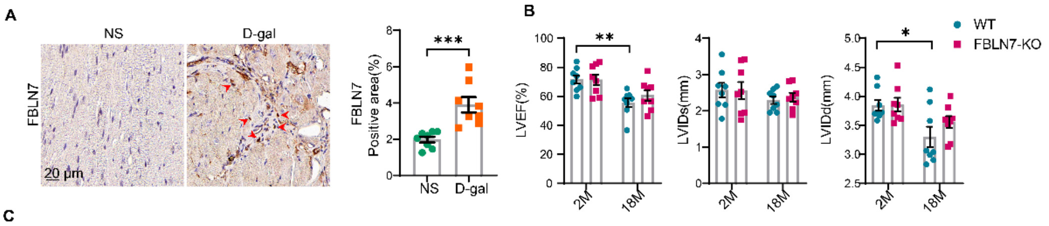

C

Tissue	Cell Type	Cell Type Full Name	Class	Gene	Avg_logFC	P_val	P_val_adj	Species	DOI
Heart	M-Fib	Myofibroblast	O vs Y.down	FBLN7	-0.371915	3.07E-07	0.00597553	Macaca fascicularis	https://doi.org/10.1038/s41422-020-00412-6
Heart	Fib	Fibroblast	O vs Y.down	FBLN7	-0.433867	2.82E-240	5.48E-236	Macaca fascicularis	https://doi.org/10.1038/s41422-020-00412-6

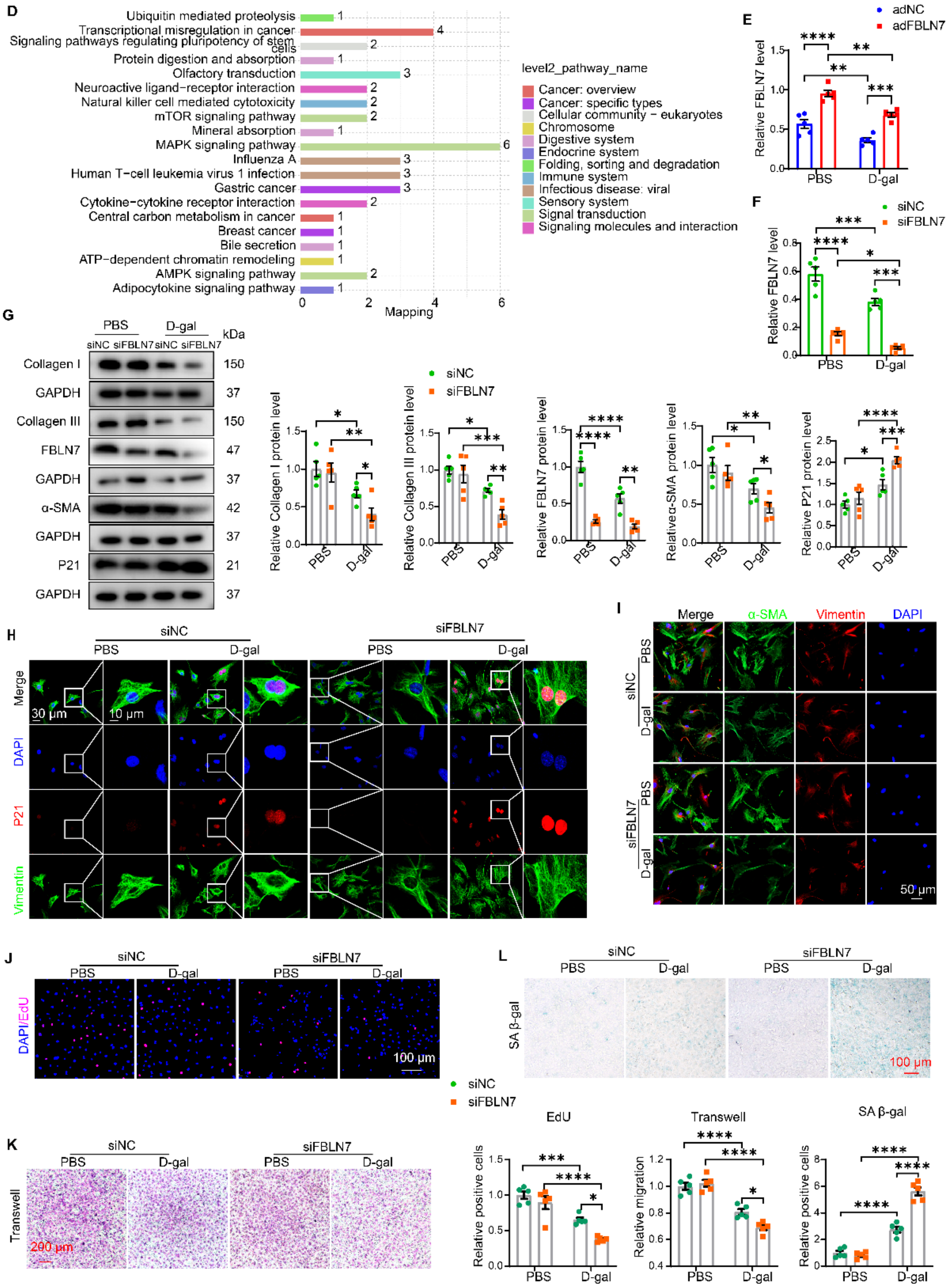

Figure S1. A) Representative images of immunohistochemical FBLN7 staining in heart sections from wild-type young mice (intraperitoneal injection of NS) and aging mice (intraperitoneal injection of D-galactose), with quantification presented on the right. B) Echocardiographic analysis of left ventricular ejection fraction (LVEF), and left ventricular internal diameter in diastole (LVIDd) and systole (LVIDs). C) The expressions of FBLN7 in cardiac fibroblasts (CFs) and myofibroblasts (MCFs) were derived from a single-cell transcriptomic atlas of primate aging hearts. The accession number for the raw snRNA-seq data is reported in the Genome Sequence Archive (GSA): CRA002689. D) Kyoto Encyclopedia of Genes and Genomes (KEGG) pathway analysis of differentially expressed proteins in the left ventricular tissues of FBLN7 gene knockout aging mice and wild-type aging mice (at 18 months old). E) The level of FBLN7 in cell culture supernatants from CFs subjected to FBLN7 overexpression after treatment with D-gal or PBS. F-L) Effects of FBLN7 silencing (siFBLN7) on D-galactose (D-gal)-induced impaired profibrotic phenotypes in senescent CFs in vitro. F) The level of FBLN7 in cell culture supernatants from CFs subjected to FBLN7 silencing after treatment with D-gal or PBS. G) Representative western blot images showing the levels of Collagen I and III, α-SMA, P21, and FBLN7 proteins in CFs subjected to FBLN7 silencing after treatment with D-gal or PBS, with quantifications displayed on the right. H) Representative images of P21 expression (red) in CFs labeled with Vimentin (green). I) Representative immunofluorescence (IF) images of CFs stained with α-SMA (green) and Vimentin (red), with nuclei stained using DAPI (blue). J) Representative images of CFs co-stained with EdU (red) and DAPI (blue), with quantification of the percentage of EdU-positive cells shown at the right. K) Representative photomicrographs from Transwell assays, with quantification of the number of migrating CFs displayed at the right. L) Representative images of senescence-associated β-galactosidase (SA β-gal) staining, along with the quantification of the relative number of SA β-gal-positive cells (blue-green) shown at the bottom. Error bars represent the standard error of the mean (SEM). Statistical significance is indicated as follows: $^*P < 0.05$, $^{**}P < 0.01$, $^{***}P < 0.001$, $^{****}P < 0.0001$.

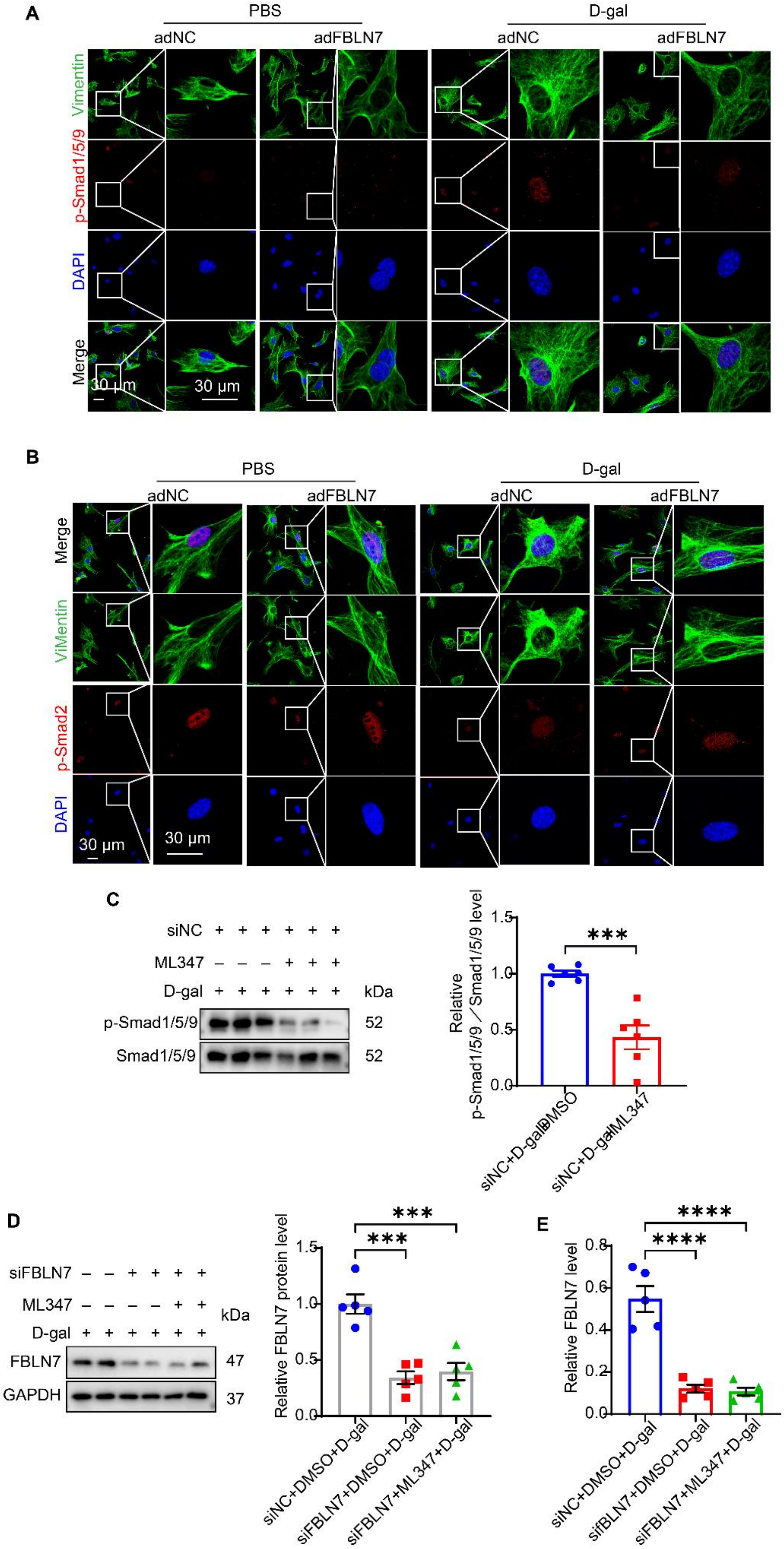
A
PBS
D-gal
adNC
adFBLN7
Vimentin
p-Smad1/5/9
DAPI
Merge
30 μm
B
Merge
ViMentin
p-Smad2
C
siNC
ML347
D-gal
kDa
p-Smad1/5/9
Smad1/5/9
52
Relative p-Smad1/5/9／Smad1/5/9 level
***
D
siFBLN7
FBLN7
GAPDH
47
37
Relative FBLN7 protein level
siNC+DMSO+D-gal
siFBLN7+DMSO+D-gal
siFBLN7+ML347+D-gal
E
Relative FBLN7 level
****

Figure S2. A-B) Representative images of p-Smad1/5/9 (red) and p-Smad2 nuclear translocation (red) in FBLN7 overexpression (adFBLN7) and control cardiac fibroblasts (adNC) after treatment with D-gal or PBS, labeled with Vimentin (green). C) Representative western blot images showing the protein levels of p-Smad1/5/9 and Smad1/5/9 in D-gal treated cardiac fibroblasts transfected with siNC treated with ML347 or DMSO. The quantification is shown on the right. D) Representative western blot images showing the protein levels of FBLN7 in D-gal treated cardiac fibroblasts transfected with siNC, siFBLN7, or siFBLN7 plus ML347 treatment. The quantification is shown on the right. Error bars represent the standard error of the mean (SEM). Statistical significance is indicated as follows: ***P < 0.001, ****P < 0.0001.

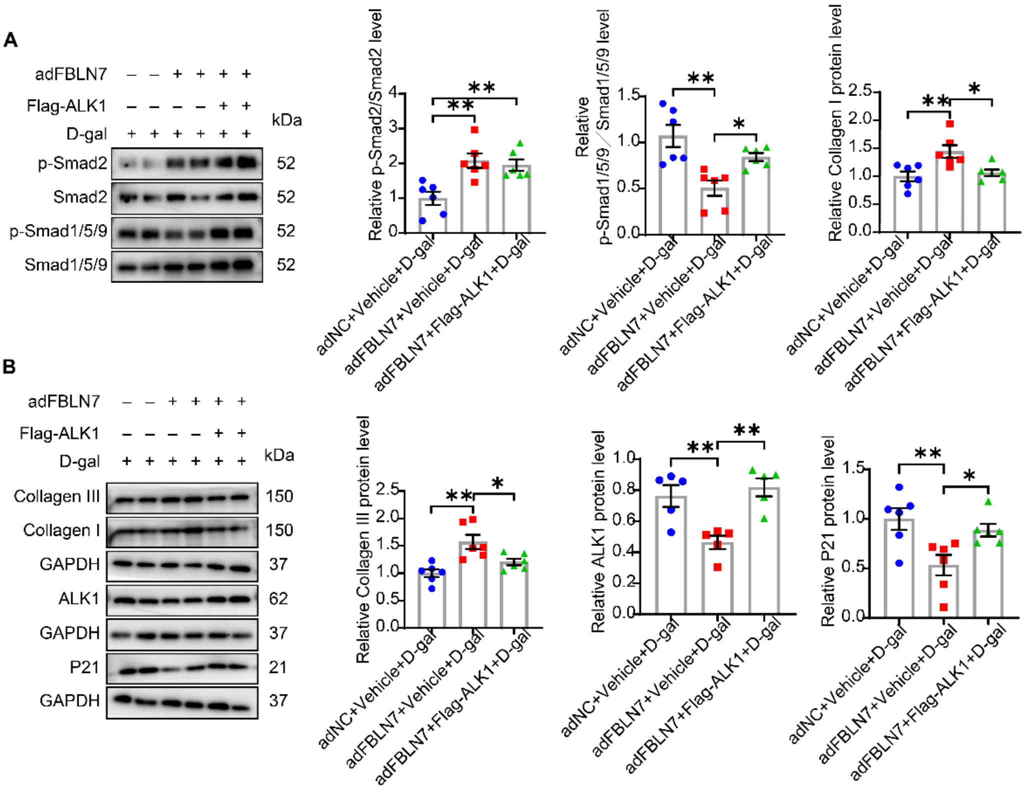

Figure S3. A) Representative western blot images illustrating protein levels of p-Smad1/5/9, Smad1/5/9, p-Smad2 and Smad2 in D-gal-treated CFs infected with either vehicle, adFBLN7, or adFBLN7 plus Flag-ALK1. Quantifications are displayed on the right. B) Representative western blot images illustrating protein levels of Collagen I and III, ALK1, and P21 in D-gal-treated CFs infected with either vehicle, adFBLN7, or adFBLN7 plus Flag-ALK1. Quantifications are displayed on the right. Error bars represent the standard error of the mean (SEM). Statistical significance is indicated as follows: *$P < 0.05$, **$P < 0.01$, ***$P < 0.001$, ****$P < 0.0001$.

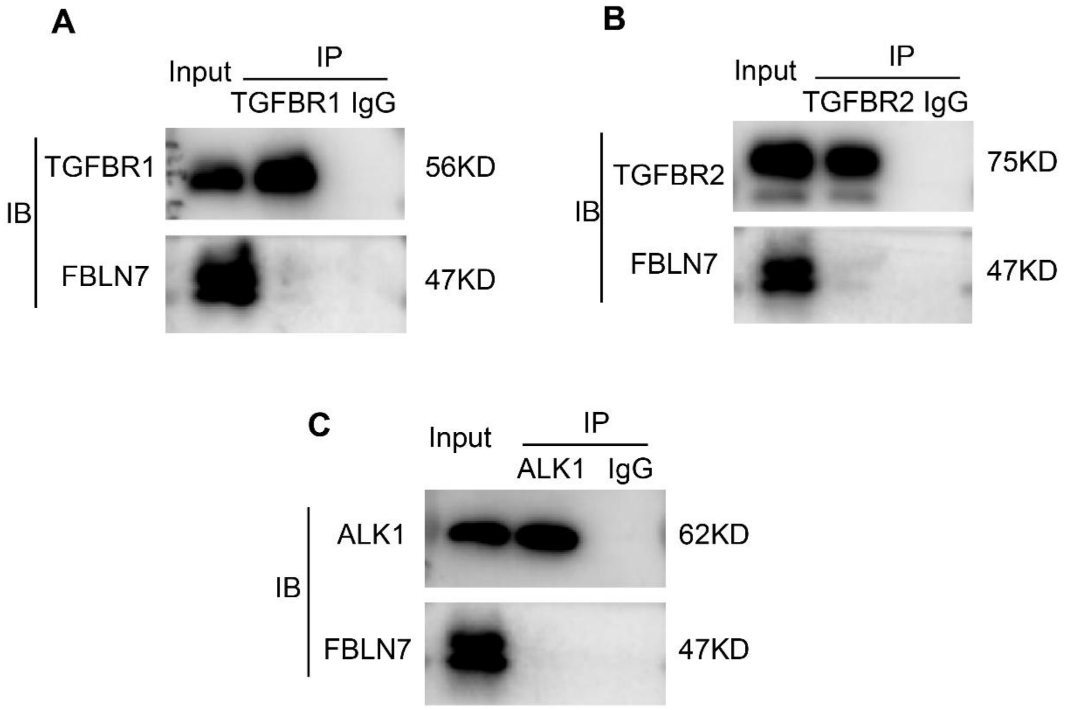

Figure S4. Endogenous co-immunoprecipitation (Co-IP) of FBLN7 with TGFBR1, TGFBR2, and ALK1. Immunoprecipitation (IP) was conducted using TGFBR1 (A), TGFBR2 (B), and ALK1 (C), followed by immunoblotting (IB) with FBLN7 in senescent cardiac fibroblasts (CFs).

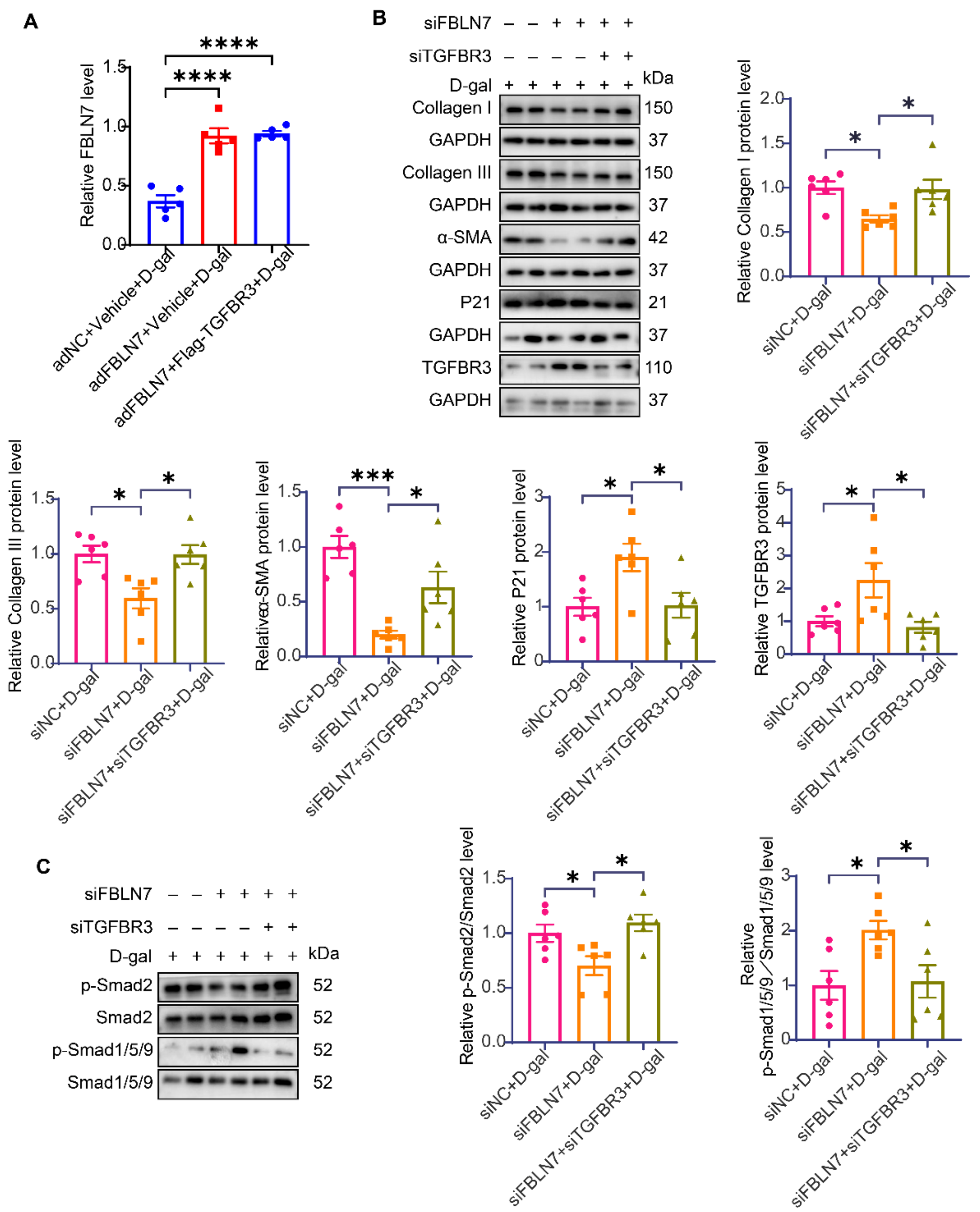

Figure S5. A) The levels of FBLN7 in cell culture supernatants from D-gal-treated CFs infected with either vehicle, adFBLN7, or adFBLN7 plus Flag-TGFBR3. B) Representative western blot images illustrating protein levels of Collagen I and III, α-SMA, P21 and TGFBR3 in D-gal-treated CFs transfected with either siNC, siFBLN7, or siFBLN7 plus siTGFBR3. Quantifications are displayed around the image. C) Representative western blot images illustrating protein levels of p-Smad1/5/9, Smad1/5/9, p-Smad2 and Smad2 in D-gal-treated CFs transfected with either siNC, siFBLN7, or siFBLN7 plus siTGFBR3. Quantifications are displayed on the right. Error bars represent the standard error of the mean (SEM). Statistical significance is indicated as follows: *$P < 0.05$, ***$P < 0.001$, ****$P < 0.0001$.

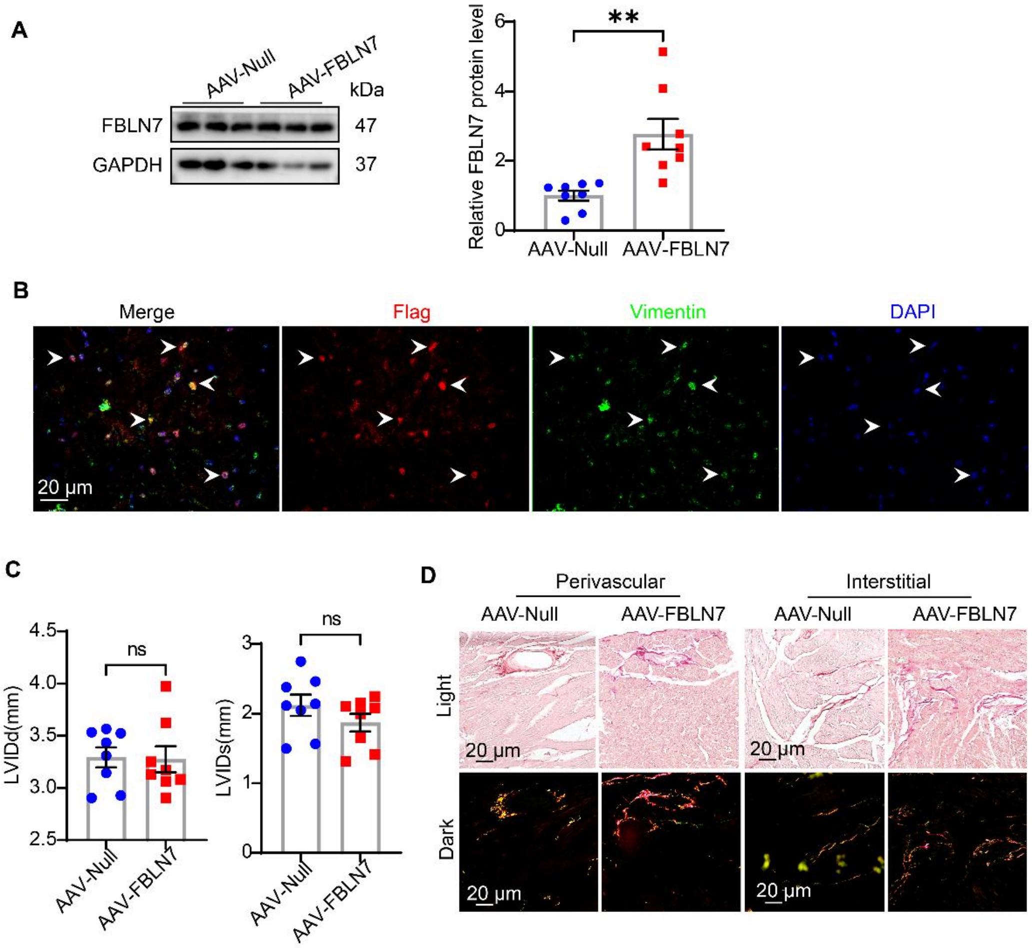

Figure S6. A) Representative western blot images displaying FBLN7 protein levels in heart tissues from mice injected with either AAV-Null or AAV-FBLN7. The quantification is presented on the right. B) Immunofluorescence staining of cardiac sections from AAV-FBLN7 mice revealed that Flag-tagged overexpressed FBLN7 co-localized with the cardiac fibroblast marker Vimentin. C) Echocardiographic analysis of left ventricular internal diameter at end-systole (LVIDs) and end-diastole (LVIDd) in aging mice (18 months old) injected with AAV-FBLN7 or AAV-Null. D) Representative Sirius red-stained heart sections from aging mice injected with AAV-FBLN7 or AAV-Null, including both perivascular and interstitial views. Error bars represent the standard error of the mean (SEM). Statistical significance is indicated as follows: $^{**}P < 0.01$, $^{***}P < 0.001$.

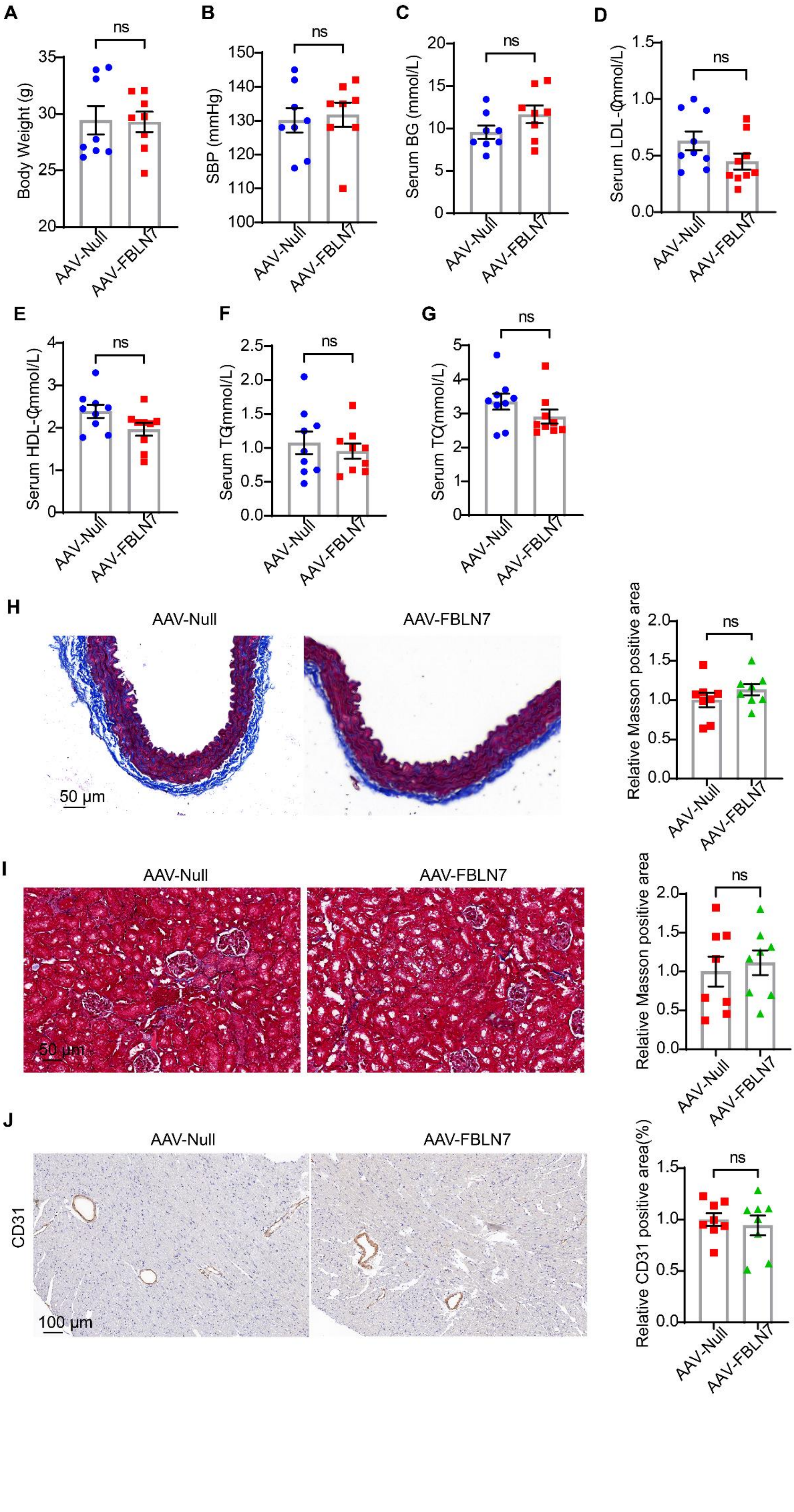
A
ns
Body Weight (g)
AAV-Null
AAV-FBLN7
B
ns
SBP (mmHg)
AAV-Null
AAV-FBLN7
C
ns
Serum BG (mmol/L)
AAV-Null
AAV-FBLN7
D
ns
Serum LDL-C(mmol/L)
AAV-Null
AAV-FBLN7
E
ns
Serum HDL-C(mmol/L)
AAV-Null
AAV-FBLN7
F
ns
Serum TG(mmol/L)
AAV-Null
AAV-FBLN7
G
ns
Serum TC(mmol/L)
AAV-Null
AAV-FBLN7
H
AAV-Null
AAV-FBLN7
50 μm
ns
Relative Masson positive area
AAV-Null
AAV-FBLN7
I
AAV-Null
AAV-FBLN7
50 μm
ns
Relative Masson positive area
AAV-Null
AAV-FBLN7
J
AAV-Null
AAV-FBLN7
CD31
100 μm
ns
Relative CD31 positive area(%)
AAV-Null
AAV-FBLN7

Figure S7. A) Results of body weight measurements in the AAV-Null and AAV-FBLN7 groups of mice. B) Results of systolic blood pressure (SBP) in the two groups. C-G) Results of metabolic indicators in the AAV-Null and AAV-FBLN7 groups: C) Comparison of blood glucose (BG); D) Comparison of serum low-density lipoprotein cholesterol (LDL-C); E) Comparison of serum high-density lipoprotein cholesterol (HDL-C); F) Comparison of serum triglycerides (TG); G) Comparison of serum total cholesterol (TC). H) Representative micrographs of Masson's trichrome staining in aortic sections from both groups of mice. The quantification is shown on the right. I) Representative micrographs of Masson's trichrome staining in kidney sections from both groups of mice. The quantification is shown on the right. J) Representative micrographs of immunohistochemical (IHC) staining for CD31 in heart sections from both groups of mice. The quantification is shown on the right. Error bars represent the standard error of the mean (SEM).

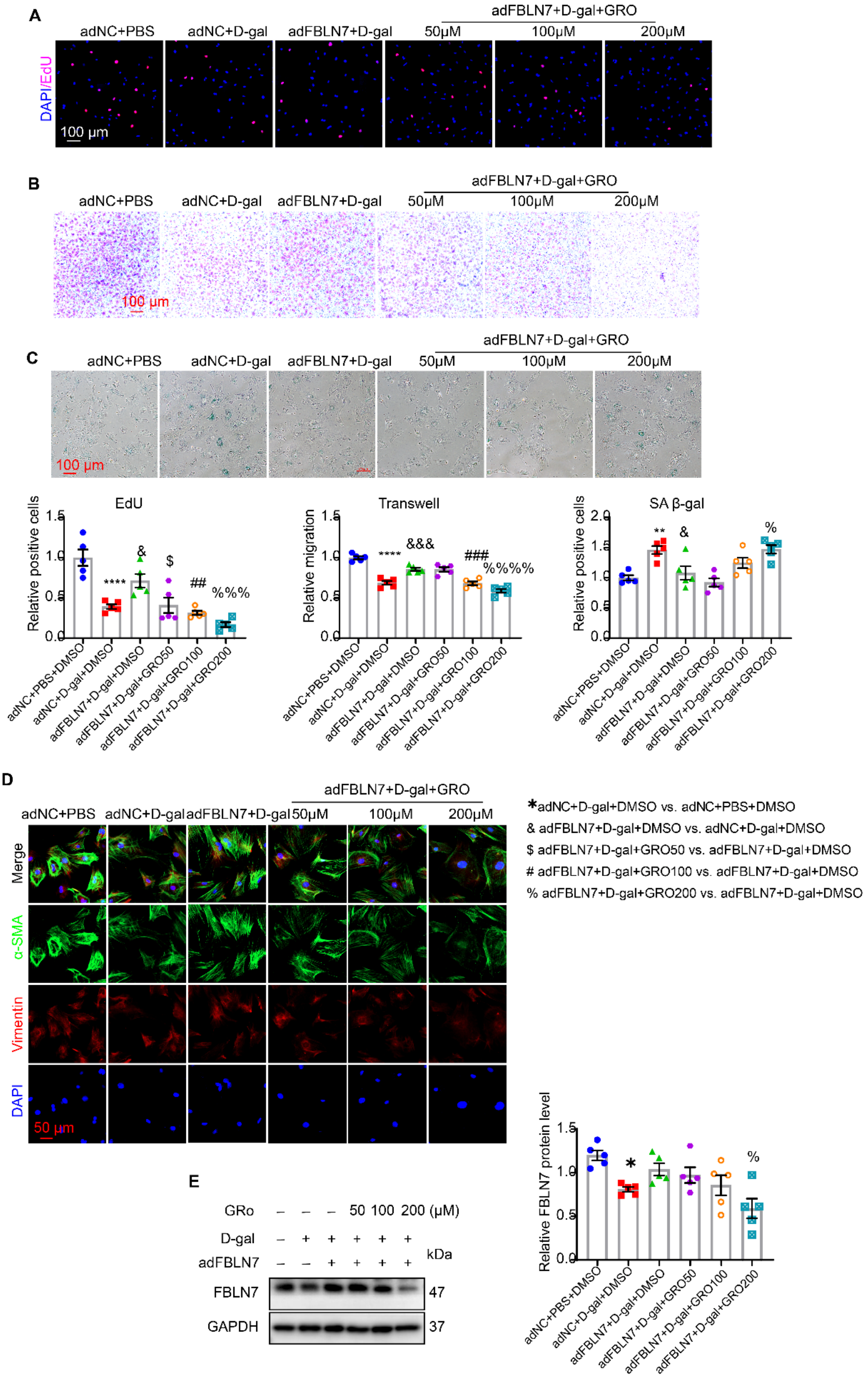

Figure S8. Effects of various concentrations of GRO treatment on the impaired profibrotic phenotypes of FBLN7-overexpressed senescent cardiac fibroblasts (CFs)

induced by D-galactose. A) Representative images of CFs co-stained with EdU (red) and DAPI (blue). The quantification is shown at the bottom. B) Representative photomicrographs of Transwell assays. The quantification of the number of migrating CFs is presented at the bottom. C) Representative images of senescence-associated β-galactosidase (SA β-gal) staining, along with the quantification of the relative number of SA β-gal-positive cells (blue-green), are displayed at the bottom. D) Representative immunofluorescence (IF) images of CFs stained with α-SMA (green) and vimentin (red), with nuclei stained with DAPI (blue). E) Representative Western blot images displaying protein levels of FBLN7 in senescent CFs infected with adenovirus encoding FBLN7 (adFBLN7) and treated with DMSO or various concentrations of GRO (50, 100, and 200 μM). The quantification is presented on the right. Error bars represent the standard error of the mean (SEM). Statistical significance is indicated as follows: $^{*}P < 0.05$, $^{**}P < 0.01$, $^{***}P < 0.001$, $^{****}P < 0.0001$.

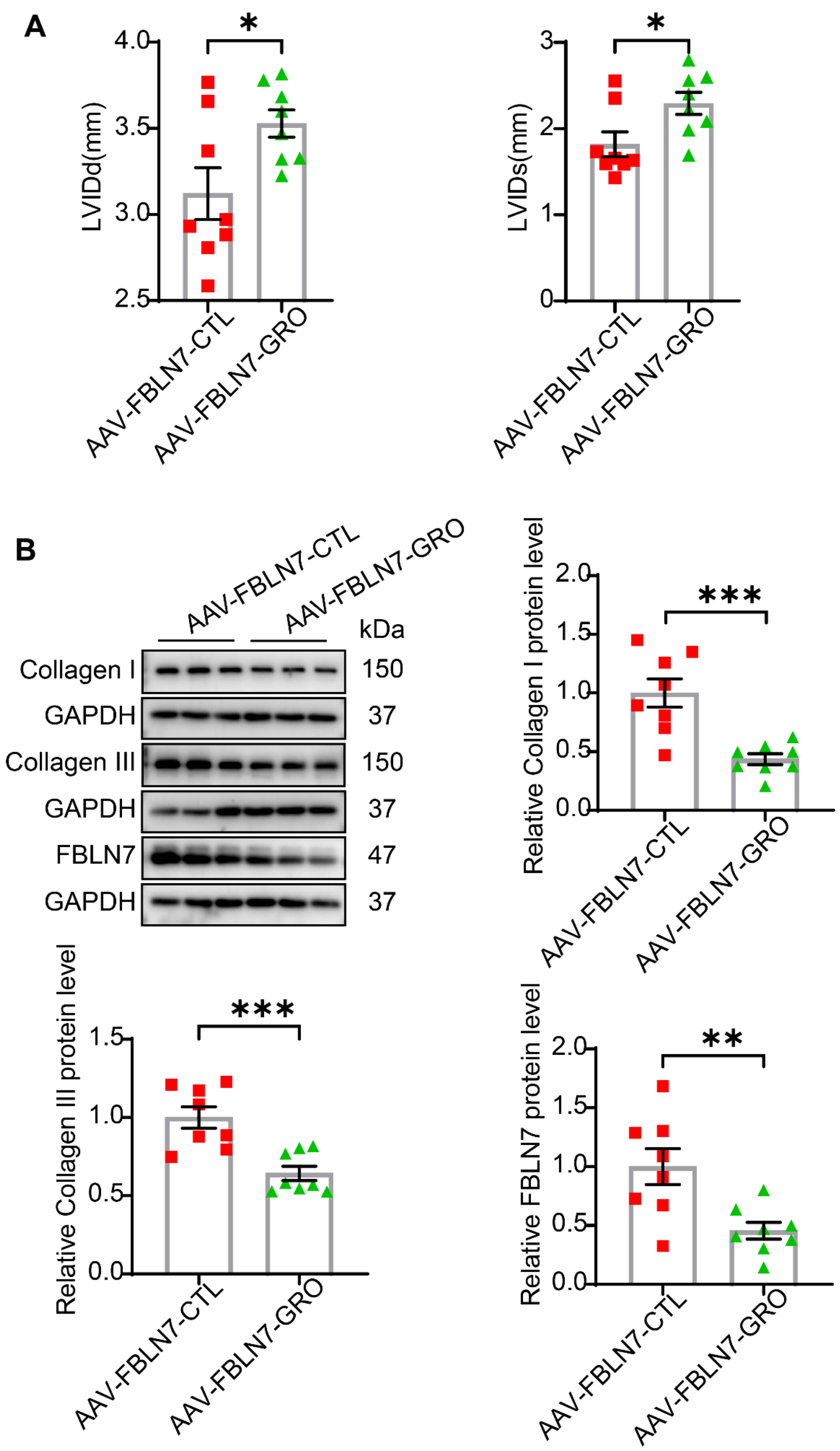
A
LVIDd(mm)
LVIDs(mm)
AAV-FBLN7-CTL
AAV-FBLN7-GRO
*
B
kDa
Collagen I
GAPDH
Collagen III
FBLN7
150
37
47
Relative Collagen I protein level
Relative Collagen III protein level
Relative FBLN7 protein level
***
**

Figure S9. A) Echocardiographic analysis of left ventricular internal diameter at end-systole (LVIDs) and end-diastole (LVIDd) in aging mice (18 months old) injected with AAV-FBLN7 treated with ginsenoside Ro (GRO) or solvent control (CTL). B) Representative western blot images displaying Collagen I, Collagen III and FBLN7 protein levels in heart tissues from AAV-FBLN7-GRO and AAV-FBLN7-CTL groups. Quantifications are presented around the images. Error bars represent the standard error of the mean (SEM). Statistical significance is indicated as follows: $^{*}P < 0.05$, $^{**}P < 0.01$, $^{***}P < 0.001$, $^{****}P < 0.0001$.

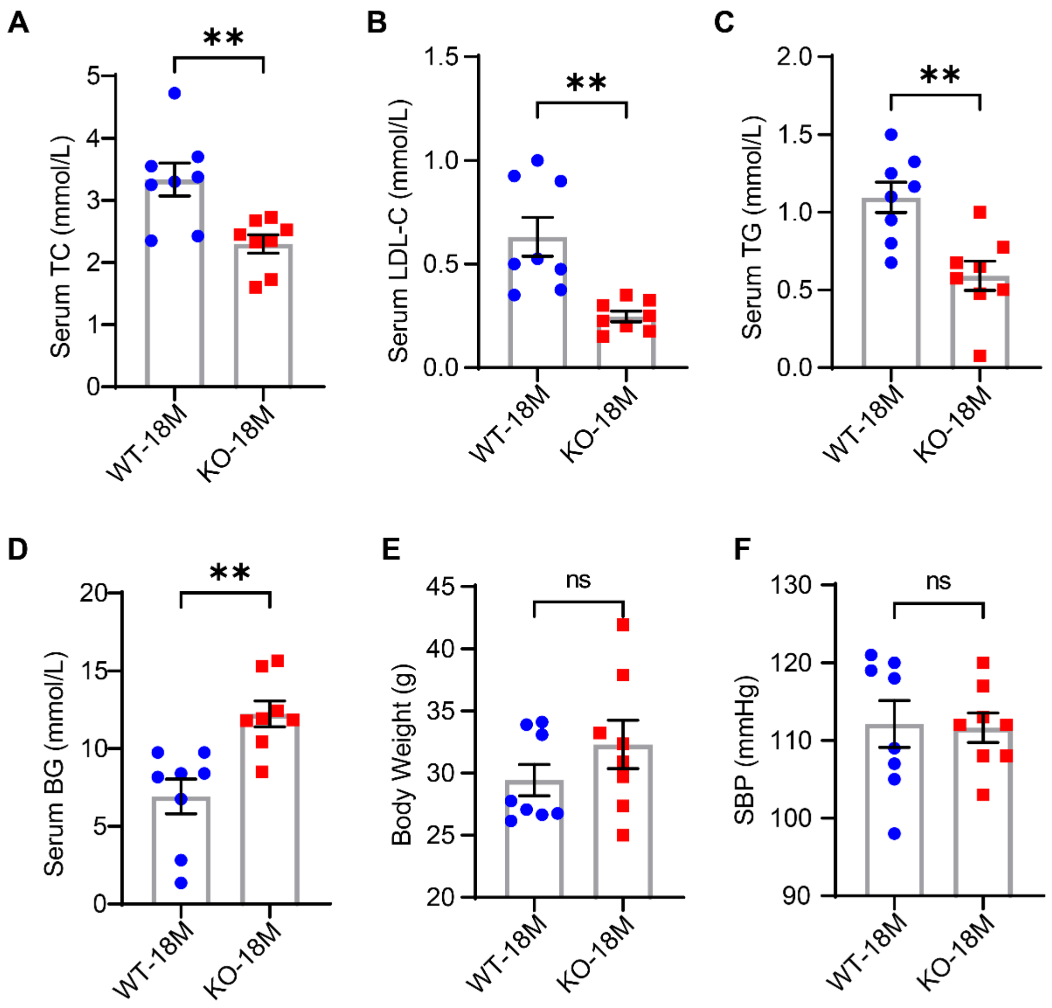

Figure S10. A-D) Results of metabolic indicators in the WT-18M and FBLN7-KO-18M groups: A) Comparison of serum total cholesterol (TC); B) Comparison of serum low-density lipoprotein cholesterol (LDL-C); C) Comparison of serum triglycerides (TG); D) Comparison of blood glucose (BG); E) Results of body weight measurements in the WT-18M and FBLN7-KO-18M groups of mice. F) Results of systolic blood pressure (SBP) in the two groups. Error bars represent the standard error of the mean (SEM). Statistical significance is indicated as follows: **$P < 0.01$.

Table S1. Antibodies and manufacturers.

Antibody	Company	Catalog No.	Concentrations
Fibulin-7	Bioss	bs-13161R	IHC1:300;<br>IF1:100
Fibulin-7	Novus	NBP2-20659	WB 1:2000
Collagen Type I	Proteintech	14695-1-AP	WB 1:2000; IHC 1:500
Collagen Type III	Proteintech	22734-1-AP	IHC 1:500
Collagen Type III	Abcam	ab184993	WB 1:1000
Collagen Type III	HUABIO	HA720050	WB 1:1000
Anti-α SMA	Abcam	ab124964	WB 1:8000
Anti-α SMA	Invitrogen	MA1-06110	IF 1:300;<br>ICC 1:500
Vimentin	CST	#5741	ICC 1:100
Vimentin	Abcam	ab8069	IF 1:200
p-Smad2 (S250)	HUABIO	ET1612-32	WB 1:2000
p-Smad2 (S250)	Immunoway	YM8030	ICC 1:100
Smad2	CST	#5339	WB 1:1000
p-Smad1(S463/S465)/5(S463/S465)/9(S465/S467)	Abcam	ab92698	WB 1:2000
p-Smad1(S463/S465)/5(S463/S465)/9(S465/S467)	Abclonal	AP1518	ICC 1:100
Smad1/5/9	Abcam	ab300164	WB 1:1000
TGFBR3	Abclonal	A0627	WB 1:1000
TGFBR3	Proteintech	20000-1-AP	WB 1:500
TGFBR3	Santa Cruz	sc-74511	ICC 1:50, IP 1:50
GFP tag	Proteintech	66002-1-Ig	WB 1:50000; IP 1:200
MYC tag	Proteintech	60003-2-Ig	WB 1:2000;<br>IP 1:200
Flag-Tag	Proteintech	20543-1-AP	WB 1:20000; IP 1:200
Cardiac Troponin I	Proteintech	21652-1-AP	IF 1:200
Anti-DDDDK tag	Abcam	Ab205606	IF 1:100
CD31	R&D Systems	AF3628	IF 1:20
CD31	Abclonal	A19014	IHC 1:500
P21	Abclonal	A19094	WB 1:500
P21	Immunoway	YM8364	ICC 1:100
TGFBR2	Abcam	ab259360	IP 1:30
TGFBR1	Abcam	ab235578	IP 1:30
